# Stratification by Mutational Landscape Reveals Differential Immune Infiltration and Predicts the Recurrence and Clinical Outcome of Cervical Cancer

**DOI:** 10.1007/s43657-024-00158-w

**Published:** 2025-03-19

**Authors:** Chun Gao, Qian Zhou, Liting Liu, Hong Liu, Yifan Yang, Shen Qu, Qing He, Yafei Huang, Ximiao He, Hui Wang

**Affiliations:** 1 Department of Obstetrics and Gynecology, Tongji Hospital, Tongji Medical College, Huazhong University of Science and Technology, Wuhan, 430030 China; 2 Cancer Biology Research Center (Key Laboratory of the Ministry of Education), Tongji Hospital, Tongji Medical College, Huazhong University of Science and Technology, Wuhan, 430030 China; 3 Department of Physiology, School of Basic Medicine, Tongji Medical College, Huazhong University of Science and Technology, Wuhan, 430030 China; 4 Center for Genomics and Proteomics Research, School of Basic Medicine, Tongji Medical College, Huazhong University of Science and Technology, Wuhan, 430030 China; 5 Hubei Key Laboratory of Drug Target Research and Pharmacodynamic Evaluation, Huazhong University of Science and Technology, Wuhan, 430030 China; 6 Department of Gynecological Oncology, Women’s Hospital, School of Medicine, Zhejiang University, Hangzhou, 310006 China; 7 Zhejiang Key Laboratory of Precision Diagnosis and Therapy for Major Gynecological Diseases, Women’s Hospital, Zhejiang University School of Medicine, Hangzhou, 310006 China; 8 Department of Pathogen Biology, School of Basic Medicine, Tongji Medical College and State Key Laboratory for Diagnosis and Treatment of Severe Zoonotic Infectious Diseases, Huazhong University of Science and Technology, Wuhan, 430030 China; 9 Department of Immunology, School of Basic Medicine, Tongji Medical College, Huazhong University of Science and Technology (HUST), Wuhan, 430030 China

**Keywords:** Cervical cancer, Recurrence, Mutational landscape, Clinical outcome, Predictive model, Immune infiltration

## Abstract

Cervical cancer (CC) is the second most common cancer of female reproductive system. However, satisfactory prognostic model for CC remains to be established. In this study, we perform whole-exome sequencing on formalin-fixed and paraffin-embedded tumor specimens extracted from 67 recurrent and 28 matched non-recurrent CC patients. As a result, four core mutated genes (i.e., *DCHS2*, *DNAH10*, *RYR1*, and *WDFY4*) that are differentially presented in recurrent and non-recurrent CC patients are screened out to construct a recurrence-free related score (RRS) model capable of predicting CC prognosis in our cohort, which is further confirmed in TCGA CESC cohort. Moreover, combining tumor mutational burden (TMB) and RRS into an integrated RRS/TMB model enables better stratification of CC patients with distinct prognosis in both cohorts. Increased infiltration of multiple immune cell types, enriched interferon signaling pathway, and elevated cytolytic activity are evident in tumors from patients with a higher RRS and/or a higher TMB. In summary, this study establishes a novel mutation-based prognostic model for CC, the predictive value of which can be attributable to immunological mechanisms. This study will provide insight into the utilization of mutational analysis in guiding therapeutic strategies for CC patients.

**Supplementary Information:**

The online version contains supplementary material available at 10.1007/s43657-024-00158-w.

## Introduction

With 604,127 new cases and 341,831 deaths estimated in 2020, cervical cancer (CC) represents the second most common cancer of female reproductive system (Sung et al. 2021). Despite the improved treatment modalities for CC, approximately 6.4% –18% of the patients with early-stage disease experience recurrence after primary treatment, and the recurrence rates further increase to 8% –40% for patients with local advanced CC (LACC) (Elit et al. 2009; Gennigens et al. 2021; Taarnhoj et al. 2018; Wang et al. 2015). Mostly detected within two years after initial treatment, recurrent CC often has a poor prognosis due to the limited treatment options (Yoshida et al. 2018). On the other hand, delayed detection of recurrence resulted from the lack of standardization in follow-up strategies, which consequently results in delayed treatment may also contribute to the poor prognosis of CC (Salani et al. 2011; Taarnhoj et al. 2018). Therefore, it is critical to predict the clinical outcome of CC in advance to carry out early intervention and improve the prognosis of CC.

Previous investigations have established that certain clinicopathological parameters (e.g., tumor stage, histologic type, tumor size, and lymphovascular space invasion [LVSI]) (Cibula et al. 2021; Paik et al. 2020; Xie et al. 2020), radiographic features (Shin et al. 2022; Zhang et al. 2022), and transcriptional signatures (Cui et al. 2022; Du et al. 2022; Wang et al. 2022; Yang et al. 2019; Yu et al. 2022) of patients at primary treatment can be used as predictive markers for CC recurrence and prognosis; however, the accuracy of these markers is not always satisfactory in clinical practice. With the rapid progress in next-generation sequencing, mutation-based biomarkers are increasingly recognized for their importance in predicting the recurrence and prognosis of a variety of cancers, given the crucial role of gene mutations in the development of cancer (Fu et al. 2023; Martinez-Jimenez et al. 2020; Tamborero et al. 2018). Another rationale of using gene mutations as biomarkers in cancer is the capacity of these mutations to generate neoantigens, thereby inducing tumor-specific immune response and leading to the regression of tumor (Robbins et al. 2013). Therefore, tumor mutational burden (TMB), initially identified as a predictor of patient response to immunotherapy (Rizvi et al. 2015; Van Allen et al. 2015), has also been used in predicting the clinical outcomes of patients with various solid tumors, including CC (Wen et al. 2021). In addition to TMB which calculates the overall number of mutations, individual gene mutations, especially those drive the development of cancer, e.g., *KRAS proto-oncogene, GTPase* (*KRAS*) (Jiang et al. 2018), *Phosphatidylinositol-4,5-Bisphosphate 3-Kinase Catalytic Subunit Alpha* (*PIK3CA*) (Chung et al. 2021), *Lysine Methyltransferase 2B* (*KMT2B*) (Li 2017)*, Erb-B2 Receptor Tyrosine Kinase 2* (*ERBB2*) (Xiang et al. 2018) and *Fibroblast Growth Factor Receptor* (*FGFR*) (Yoshimoto et al. 2020)*,* were also reported to be associated with poor prognosis of patients with CC. However, mutation-based biomarkers that can accurately predict CC prognosis are still scarce.

This study aimed to establish mutation-based predictive models for CC recurrence and prognosis. We first profiled the mutational landscape of CC patients from our cohort, based on which differentially presented mutated genes in patients with recurrent and non-recurrent CC were identified and subsequently used for constructing a recurrence-free related score (RRS) model to predict CC recurrence and prognosis. Moreover, TMB was combined with RRS to better stratification of CC patients with distinct prognosis. The prognostic value of RRS model was further evaluated in The Cancer Genome Atlas (TCGA) cohort, and were compared to that of previously reported models. Finally, the possible immunological mechanism underlying the differential prognosis of RRS- and RRS/TMB-stratified patient groups was explored through comparing different groups for their immunological characteristics. Our results will provide insight into using mutational landscape to predict CC recurrence and prognosis, and may also offer a better understanding of the underlying immunological mechanism.

## Materials and Methods

### Patients and Sample Collection

A total of 108 CC patients admitted to Tongji hospital, Hubei province of China, from January 2012 to November 2018, were enrolled in this retrospective study. The study was approved by the Medical Ethics Committee of the hospital (TJ-IRB20201208). Seventy-seven patients who met the following criteria were allocated to the recurrence group: (i) diagnosis of CC staged by the 2009 International Federation of Gynecology and Obstetrics (FIGO) staging system; (ii) tissue specimen was available; (iii) tumor recurrence was diagnosed on the basis of computed tomography scans and magnetic resonance imaging, with or without histological confirmation. Thirty-one matched patients who met the above (i) and (ii) criteria but had no tumor recurrence within three years were randomly allocated to the non-recurrence group. All patients were monitored after surgery until June 30, 2021. Disease-free survival (DFS) and Overall survival (OS) were defined as the interval between the date of surgery and the date of the first diagnosis of recurrence, and the time from the date of surgery until death or the end of follow-up, respectively. The surviving patients were censored at the end of follow-up. Formalin-fixed paraffin-embedding (FFPE) specimens from tumor and paired adjacent normal tissue of each patient were extracted from hospital archive and used for WES and Immunohistochemistry (IHC) staining.

### Study Design

As shown in Fig. S1, the study design consisted of three major steps: establishment of predictive models for CC recurrence and prognosis using mutational data extracted from our cohort, validation of the prognostic values of the models in TCGA cohort, and mechanism exploration. After initial evaluation, specimens from 67 CC patients with recurrence and 28 CC patients without recurrence were qualified for WES. Genomic and clinical data of these patients were then screened for candidate mutated genes that are closely related to clinical outcome to establish predictive models, the prognostic value of which were subsequently validated using mutational data from TCGA Cervical Squamous Cell Carcinoma and Endocervical Adenocarcinoma (CESC) patients (*N* = 289). Furthermore, tumor immune microenvironment (TIME) was examined through performing multiplex immunohistochemistry on patients' cervical tumor issue in our cohort, and by the estimation of immune-cell infiltration using RNA sequencing data from CESC patients in TCGA cohort. Genomic information of patients in our cohort are illustrated in Table S1a.

### Sample Preparation and WES Sequencing

DeoxyriboNucleic Acid (DNA) was extracted from FFPE samples from tumor tissues and paired adjacent tissues of CC patients using the QIAamp DNA FFPE Tissue Kit (QIAGEN) according to the manufacturer's instructions. DNA degradation and contamination were monitored on 1% agarose gels; and DNA concentration was measured by Qubit® DNA Assay Kit in Qubit® 3.0 Flurometer (Invitrogen, USA). A total amount of 0.2 μg DNA per sample was used as input material for DNA library preparations. Sequencing library was generated using NEB Next® Ultra™ DNA Library Prep Kit from Illumina (NEB, USA) following manufacturer's recommendations with index codes incorporated into each sample. Briefly, genomic DNA sample was fragmented by sonication to a size of 350 bp. DNA fragments were then endpolished, A-tailed, and ligated with the full-length adapter for Illumina sequencing, followed by further PCR amplification. After PCR products were purified by AMPure XP system (Beckman Coulter, Beverly, USA), DNA concentration was measured by Qubit®3.0 Flurometer (Invitrogen, USA), and libraries were analyzed for size distribution by NGS3K/Caliper and quantified by real-time PCR (3 nM).

### Mutation Calling, TMB, Tumor Purity and Mutational Signature Analyses

Adaptor and low-quality sequences were removed by the cutadapt software (Kechin et al. 2017), and the remained sequences were then aligned to human reference genome (GRCh37) using the BWA software (Li and Durbin 2009) with default parameters. SAM files were converted to BAM files followed by sorting with the Samtools software (Li et al. 2009), and duplicated sequences were marked by the Picard software. Local realignment of BAM files was performed using the GATK software (McKenna et al. 2010). Somatic mutation calling was performed by the GATK somatic MuTect2 (Cibulskis et al. 2013) with a panel of normal samples followed by filtering by Mutect2 with FilterMutectCalls. Retained somatic mutations were annotated using ANNOVAR (Wang et al. 2010). Then, high-confident somatic mutations were determined by inclusion criteria as follows: (i) somatic mutations within exon; (ii) coverage over 10 × in tumor and normal samples; (iii) variant allele frequency over 5% in tumor samples; (iv) no somatic mutation recorded in dbSNP database (Sherry et al. 2001).

TMB was computed as the number of non-silent somatic mutations per megabase. The optimal cutoff value of TMB was identified by R package survminer (Kassambara et al. 2021). And then patients were classified into the TMB^high^ and TMB^low^ groups according to the cutoff value.

Sequenza software (Favero et al. 2015) based on a probabilistic model was used to estimate tumor purity according to two major factors: a segmented average depth ratio (paired tumor *vs.* normal), and B allele frequency. Default parameters were used for tumor purity analyses.

The mutational signature of each group (e.g., RRS^high^ and RRS^low^ groups) was identified by maftools R packages as described in previous publication (Mayakonda et al. 2018).

### Construction of RRS Model

The workflow for the construction of RRS model is shown in Fig. S1a. In brief, 588 somatic mutations with frequency over 5% in our cohort were first screened by Fisher's exact test (Table S1b) and subsequently through univariate Cox regression analyses for mutated genes that are significantly differentially presented (*p* < 0.05) in the recurrence and non-recurrence groups. Then, the remaining 16 mutated genes were evaluated by least absolute shrinkage and selection operator (LASSO) Cox regression, which could not only narrow the scope of candidate genes but also prevent the model from overfitting, to optimize the selection of candidate mutated genes. This approach gave rise to 11 candidate mutated genes, which were further selected for genes with frequencies over than 5% in TCGA CESC dataset to allow broader applicability of these genes. These analyses resulted in the identification of *Dachsous Cadherin-Related 2* (*DCHS2*), *Dynein Axonemal Heavy Chain 10* (*DNAH10*), *Ryanodine Receptor 1* (*RYR1*) and *WDFY Family Member 4* (*WDFY4*) as the four core mutated genes, which were subsequently used for multivariate Cox regression analysis. Finally, the recurrence-free related score (RRS) was calculated according to the following formula:

$${\text{RRS}} = \mathop \sum \limits_{i = 1}^n b_i {\text{M}}_i ,\;M_i = \left\{ {\begin{array}{*{20}l} {1, \;\;\; {\text{if}}\; {\text{Gene }}\;i\; {\text{mutated}} } \hfill \\ {0,\;\;\; {\text{if}}\;{\text{Gene}} \;i\; {\text{not}}\; {\text{mutated}} } \hfill \\ \end{array} } \right.$$

where *b*_i_ is the Cox regression coefficient for the *i*th gene, and the selected genes (*n* = 4) and coefficients are shown in Table S2. *M*_*i*_ denotes the mutation status for the *i*th gene (*M*_*i*_ = 1 if gene *i* mutated, otherwise *M*_*i*_ = 0). Accordingly, patients were classified into high RRS subtype (RRS^high^) and low RRS subtype (RRS^low^) using the R package survminer (Kassambara et al. 2021). To choose the ideal cut-off to divide groups, the R package ‘survminer’ was used. The surv_cutpoint () function using the maximally selected rank statistics from the 'maxstat' R package, was used to split each of the sample into high- or low-RRS groups.

### Gene Expression Analysis and Cytolytic activity (CYT) Score Evaluation

Gene expression analysis was performed using gene expression data extracted from TCGA CESC cohort to determine the TIME and its association with tumor recurrence. For this purpose, previously described signature genes for various immune cells were implemented to reveal the TIME (Table S3a–c). In brief, immune cell signatures were used to establish the single-sample gene set enrichment analysis (ssGSEA) (Barbie et al. 2009) for each tumor, which could reflect the distributions of immune cells in tumor tissues. Differentially expressed genes between groups (e.g., RRS^high^ vs*.* RRS^low^) were determined using DESeq2 (Love et al. 2014), and ranked according to fold changes. ClusterProfilerR package (Yu et al. 2012) was used to visualize GSEA (Mootha et al. 2003; Subramanian et al. 2005) results based on the Molecular Signatures Database Hallmark gene set.

CYT score (Rooney et al. 2015), an index for evaluating the cytolytic activity of cytotoxic cells in tumors, was calculated as the geometric mean of *Perforin 1* (*PRF1*) and *Granzyme A* (*GZMA*) expression in transcripts per million (TPM).

### IHC

FFPE specimens were cut into 5 μm sections, on which IHC staining was performed according to the manufacturer (Proteintech, US)'s protocol (http://www.ptgcn.com/support/protocols/). Briefly, the sections were deparaffinized by dipping in xylene for 1 h and then rehydrated using gradient ethanol method. After antigen retrieval for 30 min and blocking with 3% hydrogen peroxide for 20 min, the sections were incubated overnight at 4 °C with the following primary antibodies: rabbit anti-*Signal Transducer and Activator of Transcription 1 *(*STAT1*) (clone D1K9Y, 1:1000 dilution, 14994 T, CST), and rabbit anti-*Janus Kinase 1* (*JAK1*) (clone 6G4, 1:200 dilution, 3344 T, CST). Next, samples were incubated with proper horseradish peroxidase-conjugated secondary antibodies for 1 h at room temperature followed by the application of a 3,3'-diaminobenzidine (DAB) kit to reveal the staining. Finally, the slides were scanned using the Image Viewer (version 1.1.7) and the staining intensity was measured by Image-Pro Plus6.0 (Media Cybernetics, USA).

### mIHC

Antigen retrieved FFPE sections were proceeded to multiplex immunohistochemistry (mIHC) staining using an opal 7-color manual IHC kit (Akoya biosciences, NEL811001KT), which allows simultaneous illustration of the expression pattern of up to seven antigens. The human antigen-specific primary antibodies used in this experiment were anti-CD8 (clone C8/144B, 1:5 dilution, MXB Biotechnologies), anti-CD68 (clone D4B9C, 1:8000 dilution, CST), anti-PD-L1 (clone E1L3N, 1:8000 dilution, CST), anti-PD-1 (clone EPR4877–2, 1:2000 dilution, Abcam), anti-PCK (polyclonal, 1:40 dilution, MXB Biotechnologies), anti-CD4 (clone SP35, 1:10 dilution, MXB Biotechnologies), anti-CD20 (clone L26, 1:10 dilution, MXB Biotechnologies), anti-CD56 (clone MX039, 1:10 dilution, MXB Biotechnologies), and anti-Foxp3 (clone D6O8R, 1:1000 dilution, CST). Antibodies were all validated by IHC before used for mIHC. In brief, each primary antibody specific for a given antigen (e.g., CD8) was incubated with the sections for either 30 min or overnight at optimal concentration after antigen retrieval and blocked for non-specific binding. Secondary antibody followed by TSA indirect kit were then used to amplify the signals according to the manufacturer’s instructions (Perkin Elmer). Two staining panels were designed for this study, with each panel containing six antigen/fluorescein pairs described as follows: panel 1 (CD68-Opal 520, PD-1-Opal 540, PD-L1-Opal 570, PCK-Opal 650, CD8-Opal 690 and DAPI) and panel 2 (PCK-Opal 520, CD20-Opal 540, Foxp3-Opal 570, CD4-Opal 650, CD56-Opal 690 and DAPI). Of note, six sequential procedures described above are required for each panel, after which the slides were counterstained with DAPI (Life Tech), and then covered with water-soluble anti-fluorescence quenching tablets (Cat. 0100-01, Southern Biotech). Finally, the slides were scanned using Vectra Multispectral Imaging System Version 2 (Perkin Elmer) and analyzed using the Inform software (Perkin Elmer).

### Statistical Analysis

Categorical data were analyzed using the Fisher's exact test or Chi-square test, as appropriate. Kaplan–Meier survival analyses were performed using two-sided log-rank test for revealing the difference of DFS and OS between different groups. Cox proportional hazards univariate and multivariate survival analyses were used to assess how clinical factors and Molecular characteristics predict survival. One-way ANOVA and Student’s *t-*test were used for comparing the differences between multiple groups and two groups, respectively. All statistical analyses were conducted using R software (version 4.0.2).

## Results

### Differential Mutational Landscape in Recurrent Versus Non-recurrent CC Patients

**Fig. 1:**
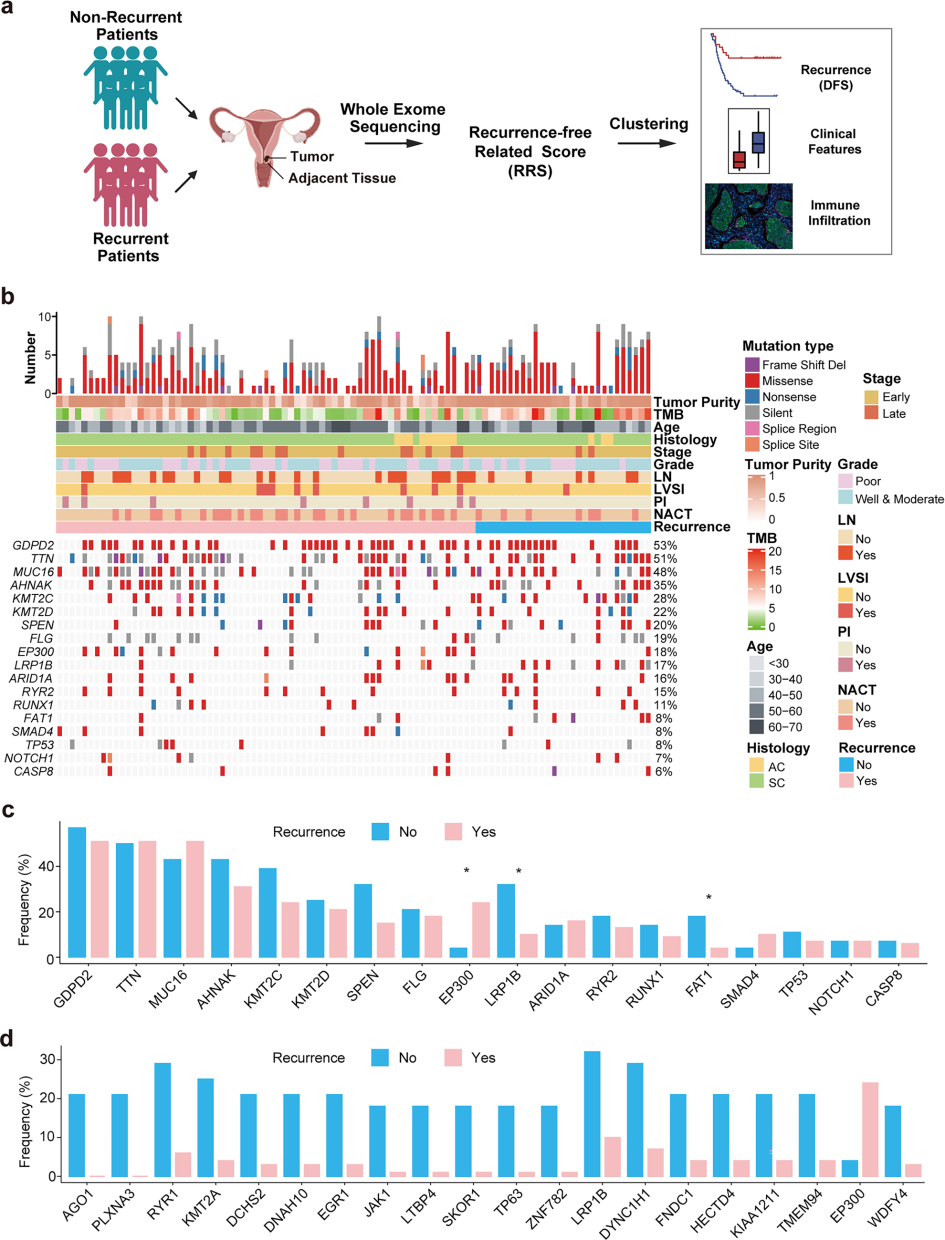
Somatic mutations in recurrence and non-recurrence CC and their associations with clinical and molecular features **a** The overall workflow and strategy for the analysis of mutational data including sample collection, experimental design and analysis. **b** Integrated plot showing the numbers and types mutations (top), clinical variables (middle) and mutations in 18 previously reported driver genes for CC (bottom) of recurrence and non-recurrence CC patients. **c** The barplot indicating the differences in the frequencies of 18 genes shown in **b** between the recurrence and non-recurrence groups. **d** The barplot indicating the top 20 mutated genes with significant difference in their frequencies between the recurrence and non-recurrence groups, and these genes are ordered by *p* values (from the minimum to maximum). Significant code * *p* < 0.05

In the quest to identify biomarkers based on mutational landscape for the prediction of CC recurrence and prognosis, 77 patients with recurrent CC and 31 matched patients without recurrence were enrolled in this study for this purpose (Table S4a, b). Initial evaluation indicated that samples from 67 recurrent patients and 28 matched non-recurrent patients were qualified for WES and mutational analysis. The clinicopathological and molecular characteristics of these patients are shown in Table 1 and Table S4c. Of note, no statistically significant differences in these parameters were found between the two groups, although patients with late-stage CC were numerically over-presented in the recurrence group compared to that in the non-recurrence group (19.4% *vs*. 7.1%, *p* = 0.217). We then sought to determine the mutational landscape of these patients for the purpose of identifying differentially presented mutated genes in the recurrence versus non-recurrence groups. The overall workflow and strategy for the analysis of mutational data are as depicted in Fig. 1a and Fig. S1. Our approach resulted in the identification of 24,216 non-silent somatic mutations including 22,374 point-mutations and 841 indels. A summary of somatic alterations is shown in Table S1a. Of note, a variety of recurrent driver mutations for CC reported previously were also found at a high frequency in our cohort: 28% for *KMT2C*, 22% for *KMT2D*, 18% for *EP300*, 16% for *ARID1A*, 8% for *FAT1*, 8% for *SMAD4*, 8% for *TP53*, 7% for *NOTCH1*, and 6% for *CASP8* (Table S5)*.* Interestingly, some mutations previously deemed as driver mutations in other solid tumors but not recurrently found in CC (Bailey et al. 2018) were also frequently observed in this cohort: 53% for *GDPD2*, 51% for *TTN*, 48% for *MUC16,* 35% for *AHNAK*, 20% for *SPEN*, 19% for *FLG*, 17% for *LRP1B*, 15% for *RYR2*, and 11% for *RUNX1* (Fig. 1b).

**Table 1:** The clinicopathological characteristics of 67 recurrent and 28 non-recurrent CC patients

Group		Overall	Non-recurrence	Recurrence	*p*-value
Total		95	28	67	
Age (median [IQR])		50.0 [45.0, 55.0]	48.0 [45.8, 55.0]	50.0 [44.5, 55.0]	0.993^a^
Stage (%)	Early (I-IIA2^b^)	80 (84.2)	26 (92.9)	54 (80.6)	0.217^c^
	Late (IIB-IV^b^)	15 (15.8)	2 (7.1)	13 (19.4)	
Histology (%)	AC	12 (12.6)	3 (10.7)	9 (13.4)	1.000^c^
	SC	83 (87.4)	25 (89.3)	58 (86.6)	
Grade (%)	Poor	40 (42.1)	9 (32.1)	31 (46.3)	0.297^d^
	Well and moderate	55 (57.9)	19 (67.9)	36 (53.7)	
LN metastasis (%)	No	64 (67.4)	22 (78.6)	42 (62.7)	0.206^d^
	Yes	31 (32.6)	6 (21.4)	25 (37.3)	
LVSI (%)	No	85 (89.5)	27 (96.4)	58 (86.6)	0.272^c^
	Yes	10 (10.5)	1 (3.6)	9 (13.4)	
PI (%)	No	87 (91.6)	28 (100.0)	59 (88.1)	0.100^c^
	Yes	8 (8.4)	0 (0.0)	8 (11.9)	
NACT (%)	No	62 (65.3)	21 (75.0)	41 (61.2)	0.293^d^
	Yes	33 (34.7)	7 (25.0)	26 (38.8)	

*AC* adenocarcinoma, *LN* lymph node involvement, *LVSI* lymph-vascular space invasion, *NACT* neoadjuvant chemotherapy, *PI* parametrial involvement, *SC* squamous carcinoma

^a^Determined by Student’s *t*-test. ^b^According to the 2009 International Federation of Gynecology and Obstetrics (FIGO) staging system. ^c^Determined by Fisher’s exact test. ^d^Determined by Chi-square test

We next compared the frequencies of mutated driver genes between the recurrence and non-recurrence groups. The results showed that the frequency of *EP300* mutation in the recurrence group was significantly higher than in the non-recurrence group, whereas the frequency of *LRP1B* and *FAT1* mutations were significantly lower in the recurrence group compared to that in the non-recurrence group (Fig. 1c). In addition to driver genes, other mutated genes were also found to be differentially presented between patients with recurrent and non-recurrent CC. The top 20 frequently mutated genes with significant differences between the two groups are shown in Fig. 1d.

### Identification of the Core Mutated Genes that are Related to the Clinical Outcomes of CC Patients

**Fig. 2:**
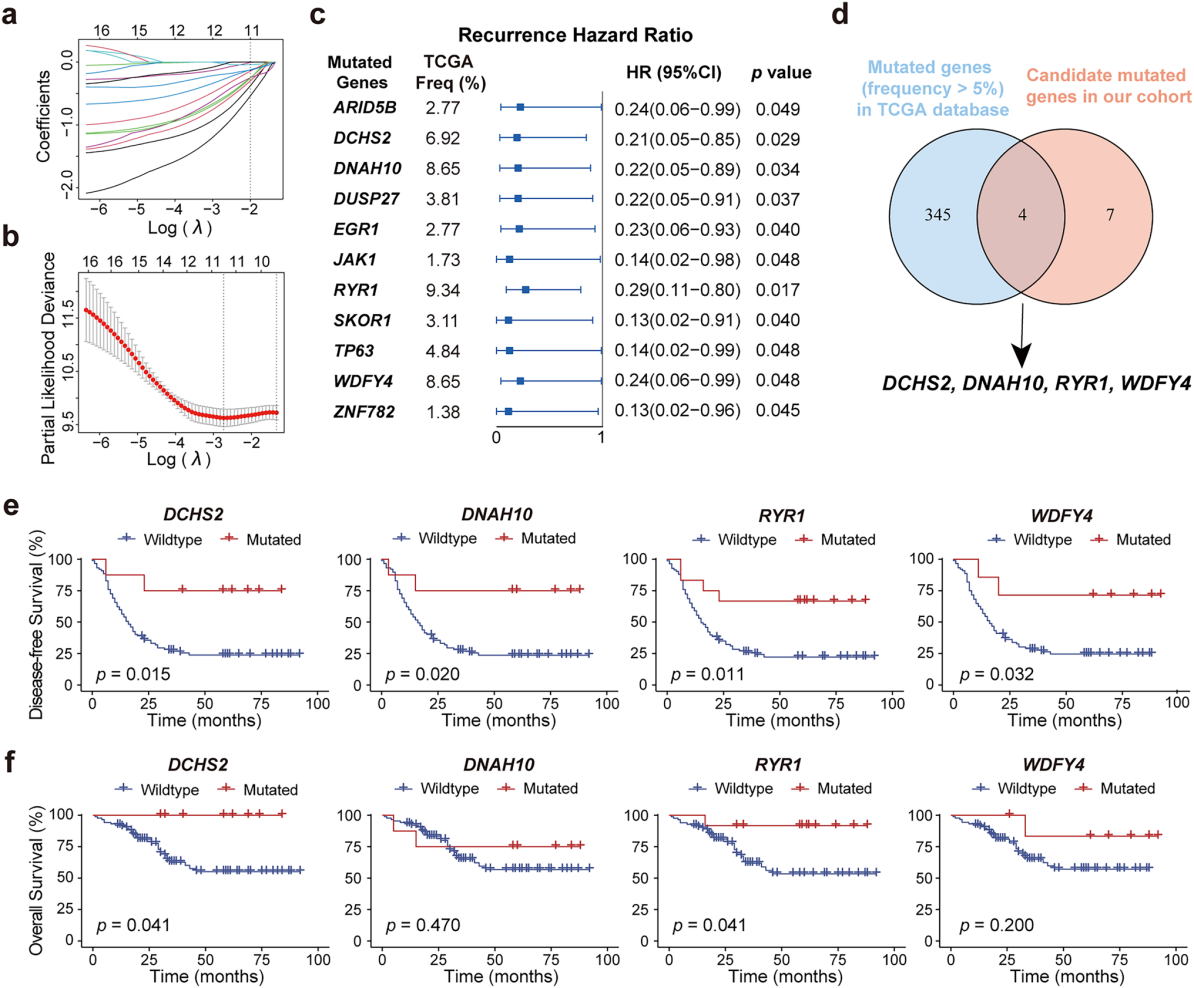
Construction of the RRS model **a** LASSO coefficient description of 11 candidate genes, with the vertical line showing the optimal value identified by tenfold cross-validation. **b** LASSO regression showing selection of optimal parameters drawn by two vertical lines, which reveal the lambda.min value (left) and the lambda.1se value (right), respectively. **c** The forest plot illustrating univariate Cox regression results of the 11 candidate genes. **d** The Venn plot showing overlapping genes between candidate genes identified in our cohort and mutated genes with frequency over 5% in TCGA CESC cohort. **e **and** f** The Kaplan–Meier survival plots showing the DFS (**e**) and OS (**f**) of patients with mutant or wildtype indicated genes

We next sought to identify the core mutated genes, regardless of being diver or not, that could be utilized to predict the clinical outcome of CC patients (Fig. S1a). In this regard, 588 mutated genes with frequencies over 5% were selected for analysis, of which 39 mutated genes that were differentially presented in the recurrence and non-recurrence groups (*p* < 0.05, Table S1b) were screened out for subsequent univariate Cox regression analysis followed by LASSO Cox regression analysis (Fig. S1b). This approach resulted in the identification of a set of 11 mutated genes with high coefficients (Fig. 2a, b). Interestingly, these 11 mutated genes are all protective factors for CC recurrence, as shown in the Fig. 2c. Next, the 11 mutated genes were further filtered by selecting mutated genes with a frequency over 5% in TCGA CESC cohort to allow a more general application. Finally, four genes, i.e., *DCHS2*, *DNAH10*, *RYR1*, and *WDFY4,* were identified as the core mutated genes (Fig. 2d).

We next asked if the four mutated genes have an impact on the clinical outcome of CC patients when examined individually. As demonstrated in Fig. 2e, patients with each of the four mutated genes had significantly better DFS than those without. When OS was analyzed, markedly better OS was similarly found in CC patients with *DCHS2* or *RYR1* mutations compared to those without, whereas no significant difference was noted between patients with or without *DNAH10* and *WDFY4* mutations (Fig. 2f).

### Establishment of a RRS Model for Predicting CC Recurrence and Prognosis

**Fig. 3:**
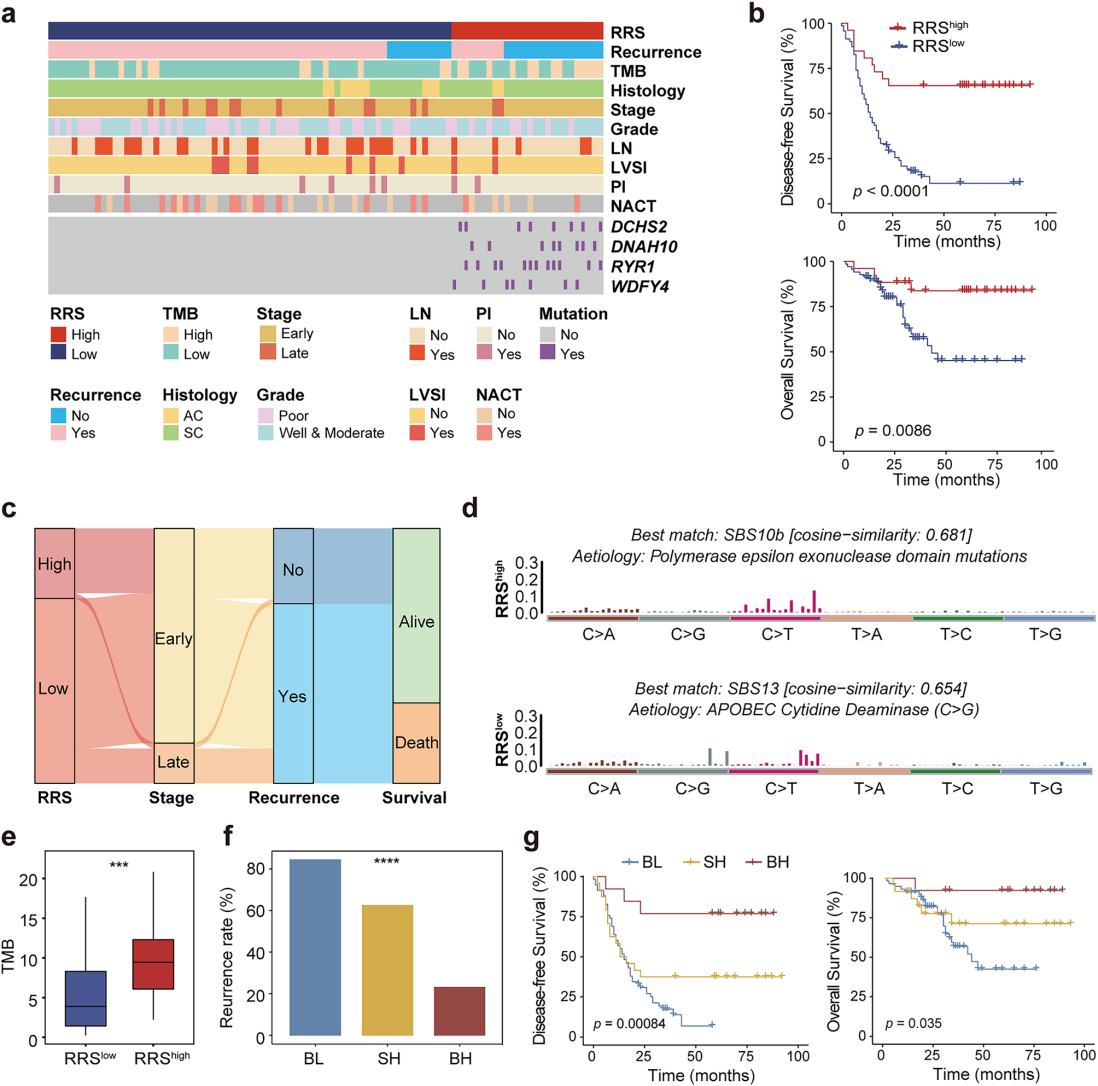
RRS is a prognostic biomarker for CC **a** Clustered heatmap of 95 samples from our cohort showing the clinical and molecular features (upper panel), and mutational status of the four core mutated genes used for constructing RRS model (bottom panel, i.e., *DCHS2*, *DNAH10*, *RYR1* and *WDFY4*) of the RRS^high^ and RRS^low^ groups. Features presented include recurrence status, histology, stage, grade, as well as status of LN, LVSI, PI, and NACT. **b** The Kaplan–Meier plot of the differences in DFS (upper panel) and OS (bottom panel) between the RRS^high^ and RRS^low^ groups. **c** The sankey diagram showing the relationship of RRS to tumor stages, as well as the status of recurrence and survival in CC. **d** The mutational signatures of the RRS^high^ and RRS^low^ groups. **e** Comparison of TMB between the RRS^high^ and RRS^low^ groups. **f** Proportion of recurrent CC patients compared among the BH, SH and SL groups. BH: both high, RRS^high^TMB^high^; SH: single high, RRS^high^TMB^low^ or RRS^low^TMB^high^; BL: both low, RRS^low^TMB.^low^. **g** The Kaplan–Meier analysis of DFS and OS for the BH, SH and SL groups. Significant code **** *p* < 0.0001; *** *p* < 0.001

The four mutated genes were mostly presented in the non-recurrence group (Fig. 3a), the correlation coefficients of the four genes and CC recurrence were all positive (Fig. 2a), and the better clinical outcomes of patients with each of the four gene mutations than those without (Fig. 2e, f), collectively suggest that these four mutated genes are candidate biomarkers indicative of the favorable prognosis of CC patients. We therefore interrogated if a combined model could be established based on these four mutated genes to accurately predict the prognosis of CC patients. For this purpose, the mutation status and coefficient of each gene were used to develop a RRS, based on which patients were classified into the RRS^high^ (*N* = 26) and RRS^low^ (*N* = 69) groups with the cutoff RRS score of 0.99 according to analysis by R package (Table S4c). In fact, patients harboring any of the four mutated genes all fell in the RRS^high^ group, which also had much higher recurrence rate (84.06%) than RRS^low^ patients (34.62%) (*p* < 0.0001; Fig. S2). Moreover, as shown in Fig. 3b and c, RRS^high^ patients had significantly better DFS and OS than RRS^low^ patients (*p* < 0.0001 and *p* = 0.0086 for DFS and OS, respectively). Importantly, these differences were even more pronounced than those between patients with and without each of the four mutated genes. Together, our results suggest that the RRS model built upon core mutated genes differentially presented in the recurrence and non-recurrence CC patients are capable of predicting the clinical outcomes of CC.

We next examined the mutational signatures of RRS^high^ and RRS^low^ patients. Our results showed that RRS^high^ patients harbored a mutational signature highly similar to SBS10B (cosine-similarity = 0.681) (Fig. 3d), which typically generates a large number of somatic mutations (> 100 mutations per MB), and samples with this signature have been designated as hypermutated (Robinson et al. 2021). In contrast, the mutational signature of RRS^low^ patients had a high similarity with SBS13 (cosine-similarity = 0.654), which is mainly C > G mutations caused by activation of *Apolipoprotein B mRNA editing enzyme, catalytic polypeptide* (*APOBEC*) cytidine deaminase and represents a characteristic mutational signature for CC. Therefore, we speculated that the difference in mutational signature between RRS^high^ and RRS^low^ CC patients may be one of the mechanisms leading to the differential clinical outcomes of these patients.

### Combination of RRS and TMB Enables Better Stratification for CC Patients with Distinct Prognosis

RRS^high^ patients had a feature of hypermutation, which often results in a high TMB. Therefore, we compared TMB in RRS^high^ and RRS^low^ patients in our cohort. As expected, RRS^high^ patients had higher TMB than RRS^low^ patients (Fig. 3e). A number of studies have reported TMB as an independent predictive biomarker for the favorable response to immunotherapy and better prognosis in a variety of cancer types, including CC (Otter et al. 2019; Sibilio et al. 2022; Sinha et al. 2022). Therefore, we examined the prognostic value of TMB alone for CC in this cohort. Our results indicated that TMB^high^ patients had significantly better DFS than TMB^low^ patients (*p* = 0.035), whereas no statistical difference was found between the two groups in terms of OS (Fig. S3a, b). These results suggest that although TMB alone can be used to predict the clinical outcomes of CC patients, its prognostic value is limited, at least in this cohort.

Given the above findings, we next explored if combining RRS with TMB (RRS/TMB) could better stratify CC patients with distinct prognosis than using RRS or TMB alone. For this purpose, patients were divided into three groups, namely BH (both high, RRS^high^TMB^high^; *N* = 13), SH (single high, RRS^high^TMB^low^ or RRS^low^TMB^high^; *N* = 24) and BL (both low, RRS^low^TMB^low^; *N* = 58). As shown in Fig. 3f, the three groups demonstrated markedly different recurrence rate (*p* < 0.0001), with the lowest being the BH group (23.08%), followed by the SH group (62.50%), whereas the BL group had the highest recurrence rate (84.48%). Moreover, DFS and OS were significantly different among the three groups (Fig. 3g; *p* = 0.00084 and *p* = 0.035 for DFS and OS, respectively). Thus, these results demonstrated that the combination of TMB and RRS has additional value in revealing the differential prognosis of more stratified CC patient subgroups.

### RRS Model Predicts CC Prognosis in TCGA CESC Cohort

**Fig. 4:**
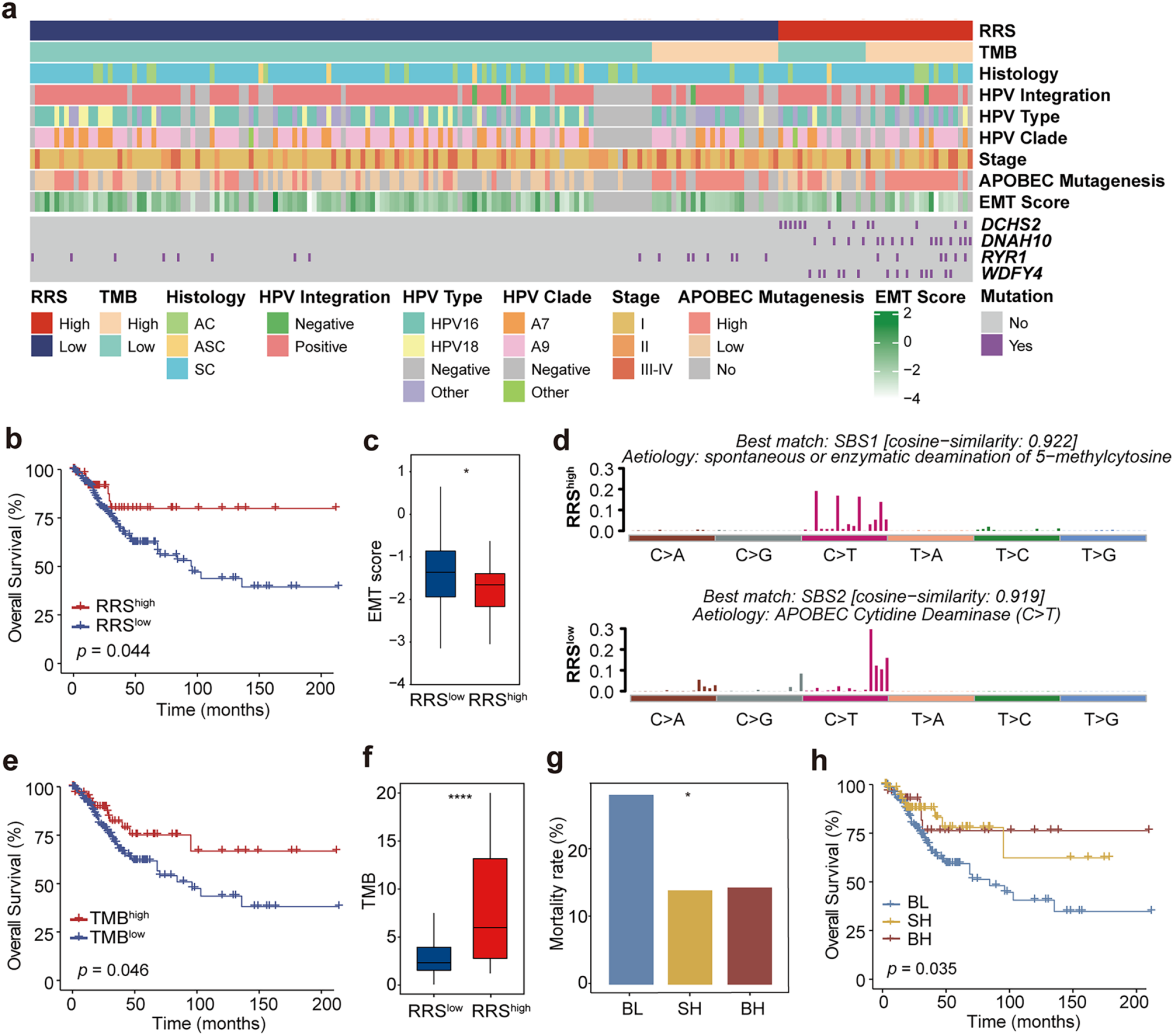
Validation of the prognostic value of RRS model in TCGA CESC cohort **a** Clustered heatmap of 289 TCGA CESC samples showing the clinical and molecular features (upper panel), and mutational status of *DCHS2*, *DNAH10*, *RYR1* and *WDFY4* (bottom) of the RRS^high^ and RRS^low^ groups. **b** The Kaplan–Meier analysis of the differences in OS between the RRS^high^ and RRS^low^ groups. **c** Comparison of EMT score between the RRS^high^ and RRS^low^ groups. **d** The mutation signatures of the RRS^high^ and RRS^low^ groups. **e** The Kaplan–Meier analysis of the differences in OS between the TMB^high^ and TMB^low^ groups. **f** Comparison of TMB between the RRS^high^ and RRS^low^ groups. **g** Comparison of mortality rate among RRS/TMB-stratified subgroups. BH: both high, RRS^high^TMB^high^; SH: single high, RRS^high^TMB^low^ or RRS^low^TMB^high^; BL: both low, RRS^low^TMB.^low^. **h** The Kaplan–Meier analysis of the differences in OS among the BH, SH and BL subgroups. Significant code **** *p* < 0.0001; * *p* < 0.05

To further evaluate the prognostic values of the RRS model for CC patients, 289 patients with CC in TCGA database were used as a validation cohort. Similar to the findings in our cohort, mutations in *DCHS2*, *DNAH10*, *RYR1* and *WDFY4* genes were also evidently concentrated in the RRS^high^ group in TCGA CESC cohort (Fig. 4a). Since the TCGA database does not include information related to CC recurrence, only OS was used for prognostic analysis. Although each of the four mutated genes was unable to differentiate the OS of CC patients (Fig. S4), high RRS was effective in identifying patients with better OS (Fig. 4b; *p* = 0.044), elaborating the findings in our cohort (Fig. 3b). Thus, these results suggest that the RRS model could be extended to other cohorts or other populations in predicting the prognosis of CC patients. We next compared other clinical variables in the RRS^high^ and RRS^low^ groups. No significant difference was found between the two groups with regard to HPV infection and integration (Fig. S5a), as well as tumor stages and histologic types (Fig. S5b, c). Interestingly, patients who did not respond to neoadjuvant chemotherapy (NACT) all had a low RRS, although the difference in NACT responsiveness between the RRS^high^ and RRS^low^ groups failed to reach statistical significance likely due to the small sample size (*p* = 0.35, Fig. S5d). Importantly, a significantly higher epithelial-mesenchymal transition (EMT) score, which is also suggestive of the poor prognosis for CC patients (Cancer Genome Atlas Research et al. 2017), was found in the RRS^low^ group compared to that in the RRS^high^ group (Fig. 4c; *p* < 0.05). Thus, the difference in EMT score, and likely NACT responsiveness may provide an explanation for the differential prognosis observed in the RRS-stratified CC patient groups.

We also examined the mutational signatures in TCGA cohort, for which the RRS^high^ group had the highest similarity with SBS1 (Fig. 4d), a signature generated spontaneously or by enzymatic deamination of 5-methylcytosine to thymine. Interestingly, similar to what we observed in our cohort, the mutational signature of the RRS^low^ group had the highest similarity with SBS2, another typical signature for CC which is mainly C > T mutations caused by APOBEC cytidine deaminase (Fig. 4d).

As observed in our cohort, RRS^high^ patients also had markedly higher TMB than RRS^low^ patients in TCGA cohort (Fig. 4e; *p* < 0.0001). On the other hand, TMB^high^ patients had significantly better OS (*p* = 0.046) than TMB^low^ patients (Fig. 4f), which is slightly different from the comparable OS between the two groups observed in our cohort (Fig. S3b). Therefore, we next questioned whether the combination of TMB with RRS are also performing better that RRS or TMB alone in TCGA cohort. Indeed, RRS/TMB combination enabled the classification of three groups, among which the mortality rate was significantly different (Fig. 4g; *p* < 0.05). More strikingly, the BH group had the best OS, followed by the SH group, while the BL group had the worst OS (Fig. 4h; *p* = 0.035). Collectively, the above findings in TCGA cohort recapitulate the added value of RRS/TMB combination revealed in our cohort.

### Differential Levels of Tumor-infiltrating Immune Cells Between RRS- and RRS/TMB-stratified Groups

**Fig. 5:**
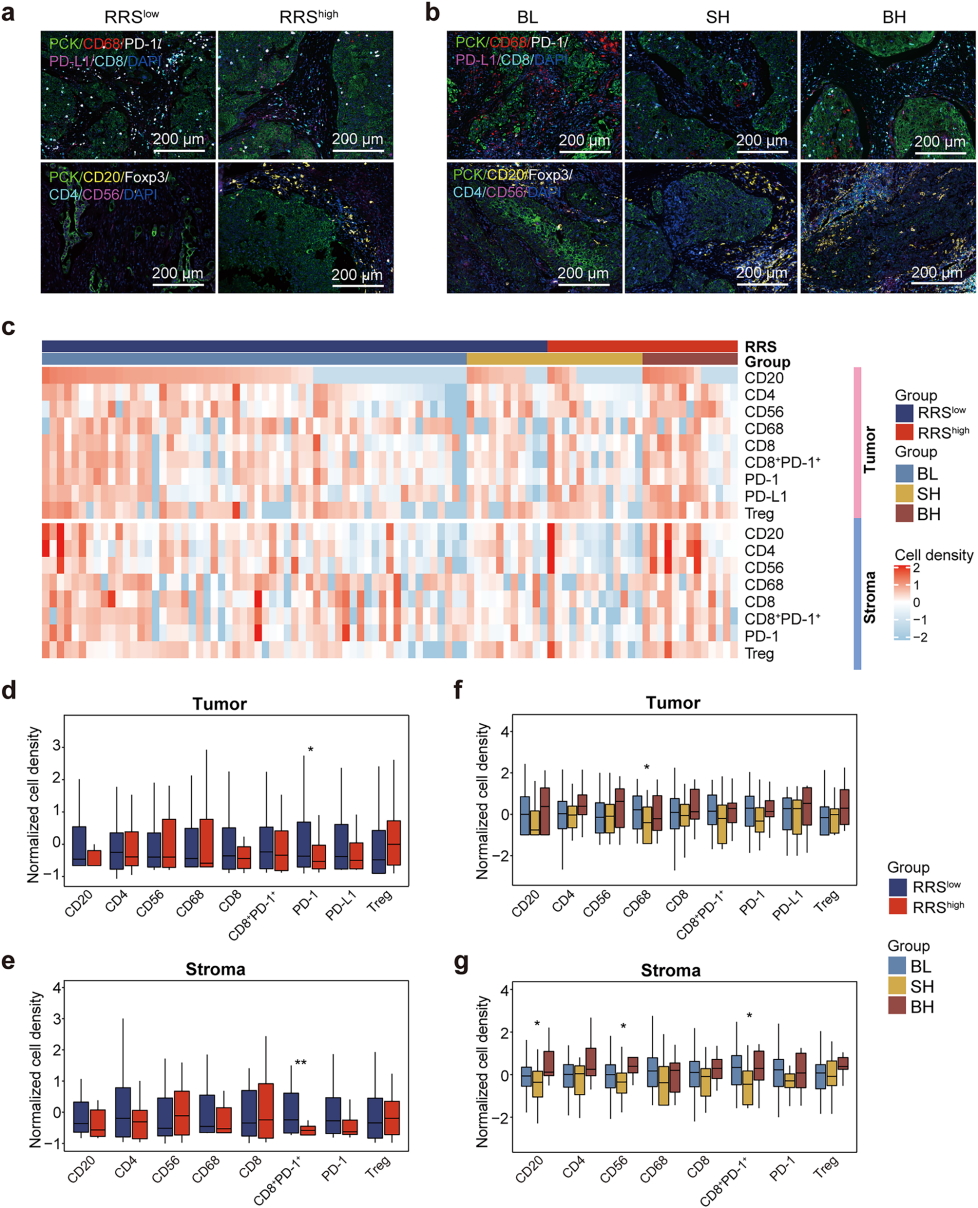
Characterization of tumor immune infiltrates in RRS- and RRS/TMB-stratified patient groups from our cohort by multiplex imaging **a **and** b** Representative mIHC images of immune cell-related markers in RRS- (**a**) and RRS/TMB-stratified (**b**) patient groups. Upper panel: PCK, CD68, PD-1, PD-L1, CD8 and DAPI; Bottom panel: PCK, CD20, Foxp3, CD4, CD56 and DAPI. **c** The heatmap showing the densities of major tumor-infiltrating immune cells in tumor center (upper panel) and stroma (bottom panel) of the RRS^high^ and RRS^low^ groups. **d **and** e** Comparison of the densities of various immune cells in tumor center (**d**) and stromal region(**e**) between the RRS^high^ and RRS.^low^ groups. **f **and** g** Comparison of the densities of various immune cells in tumor center (**f**) and stromal region (**g**) among the BH, SH and BL subgroups. Significant code ** *p* < 0.01; ** *p* < 0.05

Previous studies have shown that high tumor mutational load could give rise to the production of more tumor neoantigens, thereby inducing a more pronounced tumor-specific immune response and resulting in the regression of tumor (Ott et al. 2017). The high predictive value of the RRS model based on mutational landscape for the prognosis of CC patients, together with the implication of the *RYR1* and *WDFY4* genes used for constructing the RRS model in promoting immune response (Theisen et al. 2018; Vukcevic et al. 2013), prompted us to ask if immunological mechanisms are responsible for the differential prognosis in the RRS- and RRS/TMB-stratified groups. In this regard, mIHC was performed to illustrate the infiltration of various immune cells in the tumor center and stroma of these patients (Fig. 5a, b; Table S6). Heat maps of the density of immune cell infiltrates in RRS- and RRS/TMB-stratified groups are displayed in Fig. 5c. Comparison of immune infiltrates between the RRS^high^ and RRS^low^ groups showed that the infiltration of CD8^+^PD-1^+^ T cells was significant higher in both tumor center and stroma in the RRS^low^ group than in the RRS^high^ group (Fig. 5d, e), suggesting that exhausted PD-1^+^CD8^+^ T cells with impaired tumor-killing capacity might contribute to the worse prognosis of RRS^low^ patients.

With regard to the RRS/TMB-stratified groups, the BL group had the highest level of CD68^+^ macrophage infiltration in the tumor center among the three groups, suggesting the overall tumor-promoting effect of macrophages in this region in patients with both low RRS and TMB. In the stromal region, however, the BH group displayed the highest infiltration of CD20^+^ B cells and CD56^+^ NK cells, which were previously reported to have anti-tumor activity though antigen presentation and direct killing, respectively (Fig. 5f, g). Thus, these results indicated that the RRS/TMB-stratified groups also had differential tumor-infiltrating immune cells.

The tumor microenvironment can be divided into three different immune subtypes according to the distribution of T cells: immune-inflammatory (I-I), immune-desert (I-D), and immune-exclusion (I-E) (Binnewies et al. 2018). We therefore compared the composition of the three immune subtypes between the RRS- and RRS/TMB-stratified groups (Fig. S6a). No statistical difference was observed in the RRS^high^ versus RRS^low^ comparison (Fig. S6b). Among the RRS/TMB-stratified groups, the BH group appeared to have a higher proportion of immune-inflammatory subtype (69.23% for BH vs. 48.28% for BL, and 37.5% for SH), although the differences did not show statistical significance (*p* = 0.210) (Fig. S6c).

**Fig. 6:**
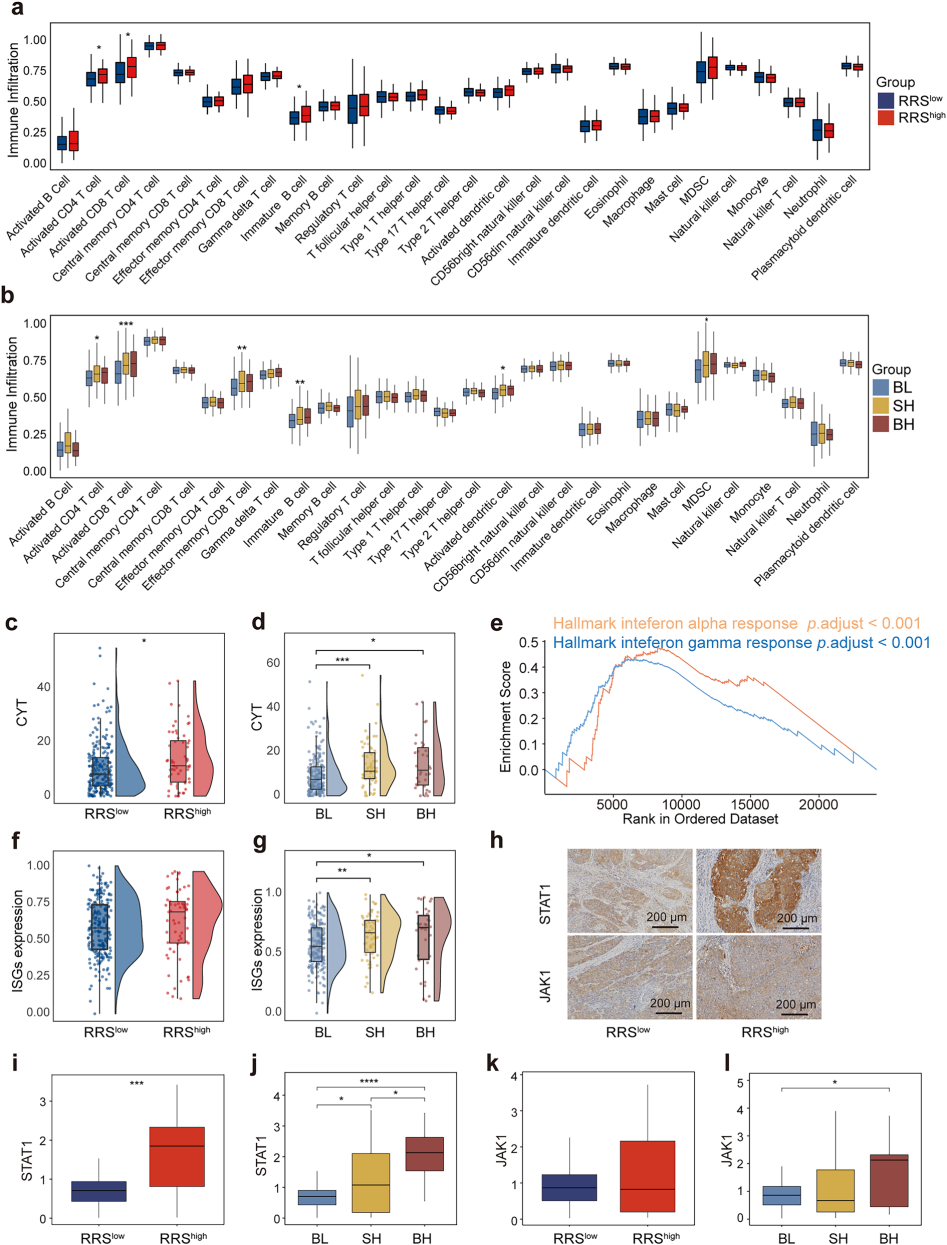
Tumor immune microenvironmental features of RRS/TMB-stratified patient groups in TCGA CESC cohort **a **and** b** Comparison of immune-cell infiltration estimated by ssGSEA analysis of transcriptome data between RRS- (**a**) and RRS/TMB-stratified (**b**) groups from 289 TCGA CESC patients. **c **and** d** Comparison of cytolytic activity scores between RRS- (**c**) and RRS/TMB-stratified (**d**) groups from TCGA CESC cohort. **e** The GSEA plot of enriched immune-related pathways in RRS^high^ patients in comparison with RRS^low^ patients. **f **and** g** Comparison of ISG scores between RRS- (**c**) and RRS/TMB-stratified (**d**) groups. **h** Representative IHC pictures of *STAT1* and *JAK1* expression in the RRS^high^ and RRS.^low^ subgroups. **i **and** j** Comparison of *STAT1* expression between RRS- (**i**) and RRS/TMB-stratified (**j**) subgroups. **k **and** l** Comparison of *JAK1* expression between RRS- (**k**) and RRS/TMB-stratified (**l**) subgroups. Significant code **** *p* < 0.0001; *** *p* < 0.001; ** *p* < 0.01; * *p* < 0.05

We next sought to confirm the above findings in TCGA CESC cohort, which only includes genome and transcriptome information instead of immunochemistry data. Nevertheless, after deconvolution of the transcriptome data with ssGSEA algorithm, we were able to evaluate more immune components and their functional status in tumor immune microenvironment, allowing the comparison for the differences in anti-tumor immune response between the RRS- and RRS/TMB-stratified groups in TCGA database. Our results showed that the infiltration of activated CD4^+^ T cells, activated CD8^+^ T cells and immature B cells was significantly higher in RRS^high^ patients than that in RRS^low^ patients (Fig. 6a). When the three RRS/TMB-stratified groups were examined, the BH and SH groups had higher infiltration of activated CD4^+^ T cells, activated CD8^+^ T cells, effector memory CD8^+^ T cells, immature B cells and activated DC cells than the BL patients, which is in line with the previously reported anti-tumor activity of these cells; however, myeloid-derived suppressor cells (MDSC), the well-described immune-suppressive cells, were also over-presented in the BH and SH groups (Fig. 6b), suggesting the complex interaction and negotiation between anti-tumor and tumor-promoting cells in the tumor microenvironment. Nevertheless, the higher infiltration of immune cells in patients with high RRS and/or high TMB demonstrated in both our cohort and TCGA cohort, may be responsible for the better prognosis in these patients.

### RRS^high^ and/or TMB^high^ Groups Exhibit Potent Cytolytic Activity and Elevated Stimulation of IFN Pathway

We next explored the possible mechanisms by which the elevated immune cell infiltration contributes to the better prognosis in patients with high RRS and/or high TMB. Firstly, CYT, a parameter indicating the tumor-killing capacity of tumor-infiltrating immune cells (Rooney et al. 2015), was compared between the RRS- and RRS/TMB-stratified groups in TCGA cohort. Our results showed that patients with a high RRS had markedly higher CYT score than those with a low RRS (Fig. 6c). Similarly, CYT scores of the BH and SH groups were also higher than that of the BL group (Fig. 6d). These results collectively suggest that high RRS and/or high TMB could result in more immune cell infiltration, which subsequently induces more pronounced tumor killing, thereby leading to the better prognosis in the RRS^high^, BH and SH groups. Next, we analyzed differentially presented signaling pathways among these groups. Our results demonstrated that the hallmarks of interferon alpha response and interferon gamma response, which are both indicative of the potency of antiviral and anti-tumor immune responses (MacMicking 2012; Parker et al. 2016), were all enriched in the RRS^high^ group (adjusted *p* < 0.001) (Fig. 6e). The above two hallmarks were also enriched in the BH and SH groups compared to that in the BL group (Fig. S7a, b). We also examined the expression of interferon-stimulated genes (ISGs). Although ISGs expression was comparable between the RRS^high^ and RRS^low^ groups (Fig. 6f), it was significantly higher in the BH and SH groups than in the BL group (Fig. 6g). Thus, elevated activation of interferon signaling pathway might also be involved in the immunological mechanisms underlying the high prognostic value of our mutational landscape-based predictive model.

*STAT1* and *JAK1* are key molecules downstream of the interferon signaling pathway (Schneider et al. 2014). Therefore, these two molecules were selected for immunohistochemical staining to verify the activation of the interferon signaling pathway at the protein level in our cohort. The results showed that the expression of *STAT1* was significantly higher in the RRS^high^ group than in the RRS^low^ group (Fig. 6h, i), as well as in the BH group than in the SH and BL groups (Fig. 6j; Fig. S7c). In addition, although there was no significant difference in *JAK1* expression between the RRS^high^ and RRS^low^ groups (Fig. 6h, k), significantly higher expression of *JAK1* was observed in the BH group versus BL group comparison (Fig. 6l; Fig. S7c). Together, these findings further highlight the importance of tumor-infiltrating immune cells, likely through the activation of the interferon signaling pathway and direct killing, for mediating the high accuracy of prognostic model based on mutational landscape.

## Discussion

Identification of predictive markers for CC recurrence and prognosis remains challenging. In this study, through analyzing the mutational landscape of a total of 67 recurrent and 28 non-recurrent CC patients, mutation-based predictive model for CC recurrence and prognosis is established and subsequently evaluated for its prognostic value in our cohort and TCGA cohort. Furthermore, the underlying immunological mechanisms are also explored. The major findings of this study can be summarized as follows: (i) recurrent and non-recurrent CC patients are significantly different in the frequencies of a number of mutated genes, including driver genes and non-driver genes for CC; (ii) four core mutated genes that were not previously identified as drive genes, i.e., *DCHS2*, *DNAH10*, *RYR1*, and *WDFY4,* are related to the clinical outcomes in our cohort; (iii) a RRS model based on the above four core mutated genes is capable of predicting CC prognosis in both our cohort and TCGA cohort; (iv) combination of RRS and TMB into an integrated RRS/TMB model allows further stratification of CC patients with distinct prognosis; (v) differential immunological features in tumor microenvironment of CC patients might contribute to the high prognostic value of our mutation-based predictive model, which has added value compared to the existing prognostic models for CC.

Somatic alterations are the cardinal feature of cancer. As one of these alterations, mutations in driver genes are regarded as the key event in the development of cancer and have been frequently used as prognostic biomarkers in an array of cancers (Stratton et al. 2009). Therefore, it is not surprising that mutations in some of these driver genes including *PIK3CA*, *KRAS*, *KMT2B, ERBB2* and *FGFR*, are also able to predict the clinical outcomes of CC, as reported by previous investigations (Chung et al. 2021; Jiang et al. 2018; Li 2017; Xiang et al. 2018; Yoshimoto et al. 2020). However, to date, the prognostic value of mutations that have not been appreciated as driver mutations has not been explored in CC. In this study, a number of these mutations are frequently observed in our cohort. Interestingly, although some driver mutations are also differentially presented in the recurrence and non-recurrence CC patients (e.g., *LRP1B,* Fig. 1c), four mutated genes that have not been recognized as diver genes previously, i.e., *DCHS2*, *DNAH10*, *RYR1*, and *WDFY4,* are screened out to be the core mutated genes capable of distinguishing the recurrence and non-recurrence groups after LASSO Cox regression analysis (Fig. 2d). In fact, these four mutated genes are able to predict the DFS of CC in our cohort when used individually (Fig. 2e). More interestingly, the RRS model which integrates the four mutated genes is more accurate in predicting the clinical outcomes of CC compared to using the four mutated genes individually, in terms of both DFS and OS (Fig. 3b, c). More importantly, RRS remains a significant prognostic biomarker (besides stage) in univariate and multivariate Cox regression (Table S7a-d). To the best of knowledge, these findings are the first to emphasize the prognostic value of mutated non-driver genes for CC.

How could a model based on mutations in non-driver genes predicts the clinic outcome of CC? Several lines of evidence suggest that the influence of these genes on anti-tumor immune response, acting either directly or indirectly, might contribute. In fact, one of the four mutated genes constituting the RRS model, *WDFY4*, has been reported to be crucial for both antiviral and anti-tumor immune responses, which is highlighted by a study showing that the loss of *WDFY4* gene results in the decreased number of CD8^+^ T cells through reactive oxygen species (ROS)-induced apoptosis (Theisen et al. 2018). In the current study, CC patients with *WDFY4* mutation have much higher *WDFY4* expression than those with wildtype *WDFY4* in TCGA cohort (Fig. S8a), suggesting that *WDFY4* mutation may promote anti-tumor immune response and the consequent better prognosis of CC through increasing the expression of *WDFY4*. Another core gene, *RYR1*, can also lead to the increased killing effect of NK cells and changes in anti-inflammatory cytokines once mutated. In addition, DC cells from mice with *RYR1* mutations outperformed those from mice with wildtype *RYR1* in their capacity to stimulate T cell proliferation and the production of natural antibody (Vukcevic et al. 2013). Thus, this evidence suggests that mutations in the core genes used in RRS model might improve the prognosis of CC through promoting anti-tumor immune response. In support of this notion, RRS^high^ patients have significantly higher infiltration of immune cells with anti-tumor activity including activated CD4^+^ T cells, activated CD8^+^ T cells and immature B cells than RRS^low^ patients in TCGA cohort, as determined by ssGSEA analysis (Fig. 6a). The accessibility to FFPE specimens in our own cohort allows us to further evaluate immune infiltration by mIHC at the protein level (Fig. 5a). Interestingly, exhausted CD8^+^PD-1^+^ cells with impaired anti-tumor activity, are significantly higher in RRS^low^ patients than in RRS^high^ patients in both tumor center and stromal region (Fig. 5d, e). The significance of this finding is twofold: First, it offers an explanation for the worse prognosis of RRS^low^ patients; Second, RRS^low^ patients might benefit from PD-1/PD-L1 blockade due to the high presence of CD8^+^PD-1^+^ cells in these patients. In addition to the quantity of tumor infiltrated immune cells, the quality of these cells may also account for the differential prognosis of RRS-stratified patient groups. In support of this assertion, CYT score, which reflects the tumor-killing capacity of immune cells, as well as the expression of genes implicated in interferon signaling pathway that indicates the potency of antiviral and anti-tumor response, are all markedly higher in the RRS^high^ group than in the RRS^low^ group in TCGA cohort (Fig. 6c, e). Moreover, the expression of *STAT1*, a key molecule downstream of the interferon signaling pathway, is also significantly higher in RRS^high^ patients than in RRS^low^ patients in our cohort, as revealed by IHC staining (Fig. 6h, i). Taken together, the prognostic value of RRS model for CC established in this study may be a result of the promoting effects of the core mutated genes used in this model on anti-tumor immune response.

Alternatively, the above core mutated genes may also influence the anti-tumor immune response and/or clinical outcome of CC indirectly through affecting the mutation status of other genes, the overall quantity of which can be calculated as TMB (Stopsack et al. 2020). In fact, prior to our current study, TMB has already been frequently used as a predictive biomarker for patient survival and treatment response to immunotherapy in a variety of solid tumors including CC (Rizvi et al. 2015; Van Allen et al. 2015; Wen et al. 2021; Zhao et al. 2021), with the mechanism involving the capability of TMB^high^ tumor in generating a high number of neoantigens, which subsequently promotes the anti-tumor immune response, thereby resulting in tumor regression and a better prognosis of cancer patients (Rizvi et al. 2015). The implication of TMB in the effect of core mutated genes on anti-tumor immune response and/or clinical outcome in CC is supported by the following evidence provided by either this study or other investigations. First, some of the four core genes are related to a high TMB in an array of cancers including CC. A study based on small cell lung cancer showed that patients with mutations in *DNAH10* (one of the four core genes) has a higher tumor mutation load (Li et al. 2020). Another core mutated gene, *DCHS2*, which plays an important role in cell adhesion and polarization, was reported as the main molecular feature of high microsatellite unstable gastric cancer and colorectal cancer, which are well-known for their capacity to generate a high TMB (An et al. 2015). In this study, three of the four core mutated genes (i.e., *DCHS2*, *DNAH10*, *RYR1*) are all significantly correlated with a higher TMB in our cohort (Fig. S8b). Second, the mutational signature of RRS^high^ patients in our cohort is highly similar to SBS10B (Fig. 3d), which is a typical signature with the capacity to generate a high TMB (Robinson et al. 2021); Finally, RRS^high^ patients have markedly higher TMB than RRS^low^ patients in both our cohort (Fig. 3e) and TCGA cohort (Fig. 4f). On the other hand, TMB^high^ patients have a better OS than TMB^low^ patients in TCGA cohort (Fig. 4b); and the TMB^high^ patients also have a better DFS and a trend toward longer OS than TMB^low^ patients in our own cohort, although the difference in OS is not statistically significant (Fig. S3a, b). Thus, high TMB could be another mechanism responsible for the better clinical outcomes in RRS^high^ patients.

As discussed above, RRS and TMB may represent two inter-connected but non-redundant biomarkers for the prediction of CC prognosis. The above notion, therefore, provides the rationale of combining both parameters to provide a better stratification of CC patients with differential prognosis. Indeed, RRS/TMB combination is capable of revealing the differential prognosis of more stratified CC patient subgroups, i.e., BH, SH and BL. Specifically, the BH group has the best clinical outcomes, followed by the SH group, and finally the BL group in both our cohort (Fig. 3g) and TCGA cohort (Fig. 4h). Strikingly, the biggest difference lies between the BH and BL groups, indicating the robustness of this strategy in separating patients with the best and worst prognosis. Interestingly, the SH group, in which patients are either RRS^high^TMB^low^ and RRS^low^TMB^high^, has an intermediate prognosis. This finding demonstrates the advantage of RRS/TMB combination in identifying an additional patient group with distinct prognosis that might be masked by classifications using RRS or TMB alone, and also suggests that tailored treatment options could be applied to these RRS/TMB-stratified patient groups to improve the prognosis of CC.

Furthermore, the BH and SH groups have higher infiltration of anti-tumor immune cells including activated CD4^+^ T cells, activated CD8^+^ T cells, effector memory CD8^+^ T cells, immature B cells and activated DC cells than the BL patients in TCGA cohort (Fig. 6b), as evaluated at the transcriptional level, suggesting that differential anti-tumor immune responses might also contribute to superior capacity of RRS/TMB combination to stratify CC patients with distinct prognosis. Similar findings are recapitulated in our cohort by IHC staining, in which the tumor-killing CD56^+^ NK cells and antigen-presenting CD20^+^ B cells are also over-presented in tumor stroma in the BH group (Fig. 5f, g). However, in TCGA cohort, the increase of aforementioned anti-tumor immune cell subsets is accompanied by the elevation of MDSC (Fig. 6b), a well-known tumor-promoting cell population that has also been found to be associated with poor prognosis of CC (Anwaier et al. 2022; Kawano et al. 2015; Mabuchi et al. 2014; Sieminska and Baran 2022; Yokoi et al. 2019), although this result cannot be validated by IHC in our cohort due to the heterogeneity of MDSC and the lack of specific markers for this cell population (Bronte et al. 2016; Khan et al. 2020). Nevertheless, the above seemingly contradictory findings in TCGA cohort may reflect the complex cross-regulation between anti-tumor and pro-tumor immune cells in tumor microenvironment during disease development, while the abundance of MDSC in the BH and SH groups suggests that the prognosis of these patients could be further improved by therapeutic strategies counteracting MDSC through inhibiting the infiltration, expansion, differentiation and functionality of MDSC, as suggested by recent reviews (Sieminska and Baran 2022; Veglia et al. 2021). In addition to the differential presence of infiltrated immune cell subsets, the qualities of anti-tumor immune response are also significantly different among RRS/TMB-stratified patient groups. In TCGA cohort, the CYT score and ISGs expression are both higher in the BH and SH groups than in the BL group (Fig. 6d, g), and the hallmarks of interferon alpha response and interferon gamma response are also enriched in the BH and SH groups compared to the BL group (Fig. S7a, b). Moreover, in our cohort, the expression of *JAK1* at the protein level is higher in the BH and SH groups than in the BL group, while *STAT1* expression is higher in the BH group compared to that in the SH and BL groups (Fig. 6h, j). Collectively, the evidence provided above emphasizes the advantage of the combination of RRS and TMB in identifying more patient groups with differential prognosis, to which the distinct quantity and quality of anti-tumor immune response in different patient groups might contribute.

Do these advantages provide our model an added value when compared to the existing prognostic models for CC? Comparisons between different prognostic models are notoriously difficult for a number of practical reasons including the unavailability of a third-party dataset that allows this type of comparison, and the possibility that certain parameters used in one model might be absent in other models. Nevertheless, we have examined the existing prognostic models for CC published in recent years, most of which were built upon either differentially expressed genes measured by RNA-seq or microarray (Cui et al. 2022; Du et al. 2022; Yang et al. 2019; Yu et al. 2022), or the clinical characteristics of cervical cancer patients (Cibula et al. 2021). Area under the curve (AUC) of above models and ours was, therefore, determined and compared. As shown in Table S8, the 1-year, 3- and 5-year AUC of the current study are comparable to (Du et al. 2022), or even better (Cibula et al. 2021; Cui et al. 2022; Wang et al. 2022; Yu et al. 2022) than the published prognostic models for CC, although they were constructed on distinct parameters and trained in different datasets. In another investigation published by Tian et al. (Tian et al. 2021), significant mutated genes (SMGs) of circulating tumor DNA (ctDNA) were used to construct a prognostic model for CC. Interestingly, their model was trained on their own dataset, which is similar to ours, allowing a direct comparison of these two models in an independent data set, i.e., TCGA data set. As shown in Fig. S9, neither the single mutated gene (Fig. S9a-e) nor the combined SMG (Fig. S9f) used by Tian et al. (2021) can stratify the prognosis of cervical cancer patients in the TCGA cohort. While both the RRS model (Fig. 4b) and RRS/TMB combination (Fig. 4h) in our manuscript can stratify the prognosis of patients with cervical cancer in TCGA. Therefore, the RRS model does have an added value in predicting CC prognosis despite the existence of other prognostic models.

In summary, our RRS model is the first prognostic model based solely on gene mutation information identified by whole exome sequencing. The added value of our models may result from direct and indirect effects of the core mutated genes on anti-tumor immune response. In addition, to the best of our knowledge, the current study provides by far the largest WES dataset of recurrent CC, which will be available for other researchers and therefore a valuable source for the CC research community.

There are some limitations to our study. First, the unavailability of protein expression data in TCGA cohort and the lack of RNA-seq data in our cohort due to RNA degradation of the aged FFPE samples do not permit the direct comparison between findings in the two cohorts at the same level. Nevertheless, the effectiveness of the aforementioned prognostic model established upon our cohort is also valid when tested in TCGA cohort. Second, this retrospective study has a relatively small sample size and was conducted in only one center, thus prevents us from drawing a definitive conclusion regarding the robustness of our prognostic model. Finally, FFPE samples are used for WES detection and subsequent mutational analysis; however, due to the age of some samples, the depth of sequencing data may be limited, as reported previously (De Paoli-Iseppi et al. 2016). Therefore, prospective investigations including more patients with available fresh-frozen tumor samples are required to further consolidate the prognostic value of the RRS model.

## Conclusion

Despite these limitations, the current study is the first to construct prognostic model for CC based upon mutations in non-driver genes, to the best of our knowledge. The resulting RRS model is effective in predicting CC prognosis both in our cohort and TCGA cohort. Moreover, the combination of RRS and TMB could identify more stratified patient groups with differential prognosis. Thus, the above findings, together with the differential anti-tumor immune responses in RRS- and RRS/TMB-stratified patient groups will shed light on the accurate prediction of CC prognosis and accordingly more tailored treatment strategies for CC in the future.

## Supplementary Information

Below is the link to the electronic supplementary material.

Supplementary file1 (TIF 11994 KB)

Supplementary file2 (TIF 947 KB)

Supplementary file3 (TIF 2040 KB)

Supplementary file4 (TIF 2155 KB)

Supplementary file5 (TIF 7395 KB)

Supplementary file6 (TIF 8986 KB)

Supplementary file7 (TIF 4959 KB)

Supplementary file8 (TIF 6829 KB)

Supplementary file9 (TIF 5989 KB)

Supplementary file10 (XLSX 7972 KB)

Supplementary file11 (XLSX 16 KB)

Supplementary file12 (XLSX 278 KB)

Supplementary file13 (XLSX 32 KB)

Supplementary file14 (XLSX 16 KB)

Supplementary file15 (XLSX 28 KB)

Supplementary file16 (XLSX 20 KB)

Supplementary file17 (XLSX 16 KB)

## Data Availability

All data needed to evaluate the conclusions in the paper are present in the paper and Additional files. The raw WES data are stored in the National Genomics Data Center (https://ngdc.cncb.ac.cn/) with the accession number CRA007769 (https://ngdc.cncb.ac.cn/gsa/s/71og2zo7). Other data and materials are available upon reasonable request to corresponding authors.
