# Supplementary figures and images for: Stratification by Mutational Landscape Reveals Differential Immune Infiltration and Predicts the Recurrence and Clinical Outcome of Cervical Cancer
Source: Phenomics. 2025 Mar 19;5(4):384–403. doi: 10.1007/s43657-024-00158-w (PMC12457260; doi:10.1007/s43657-024-00158-w)

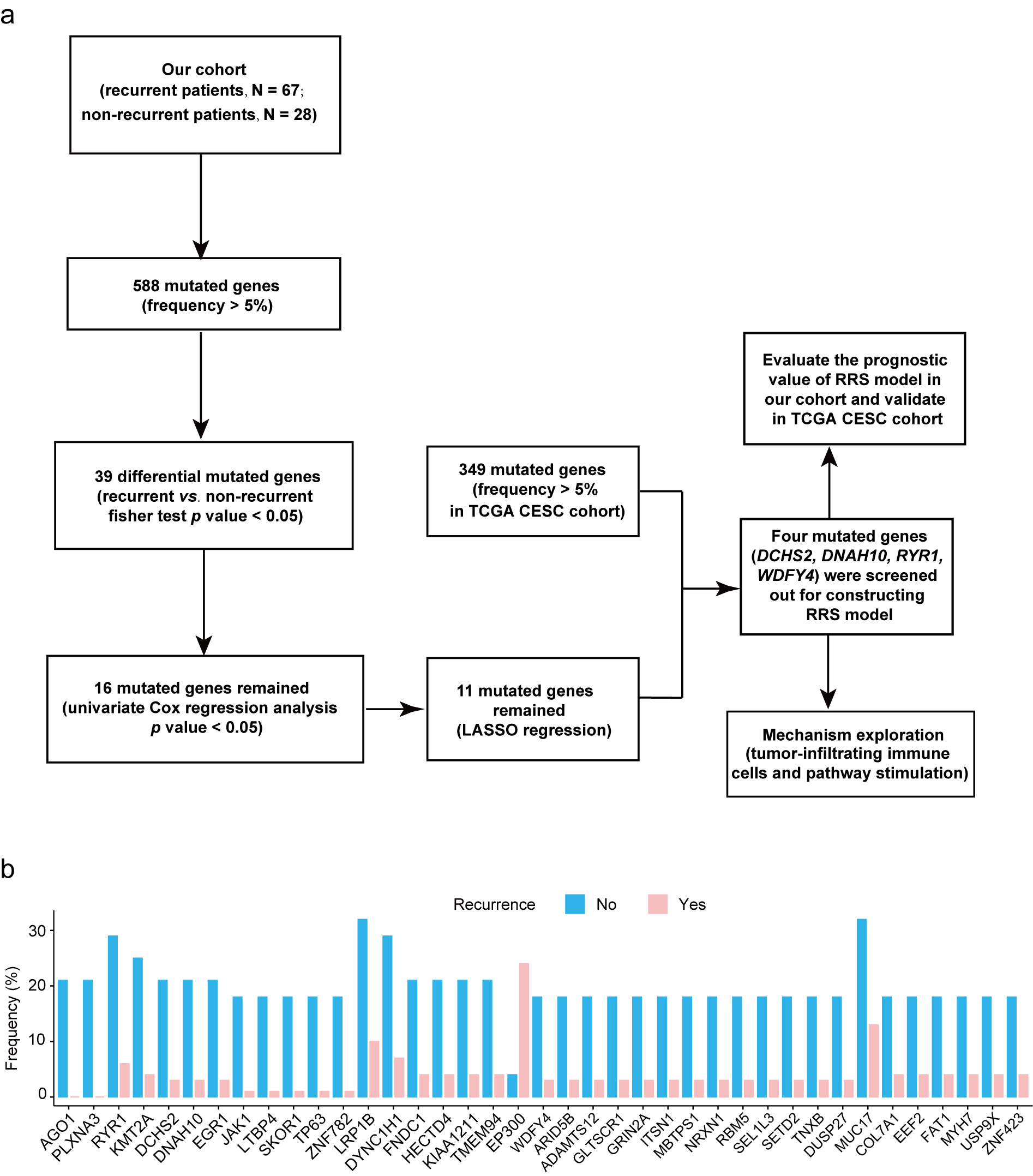

Supplement: Supplementary file 1 — Supplementary file1 (TIF 11994 KB) [file 43657_2024_158_MOESM1_ESM.tif]

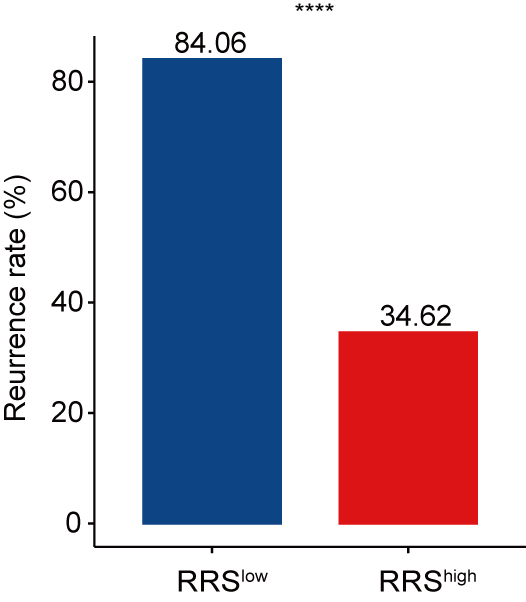

Supplement: Supplementary file 2 — Supplementary file2 (TIF 947 KB) [file 43657_2024_158_MOESM2_ESM.tif]

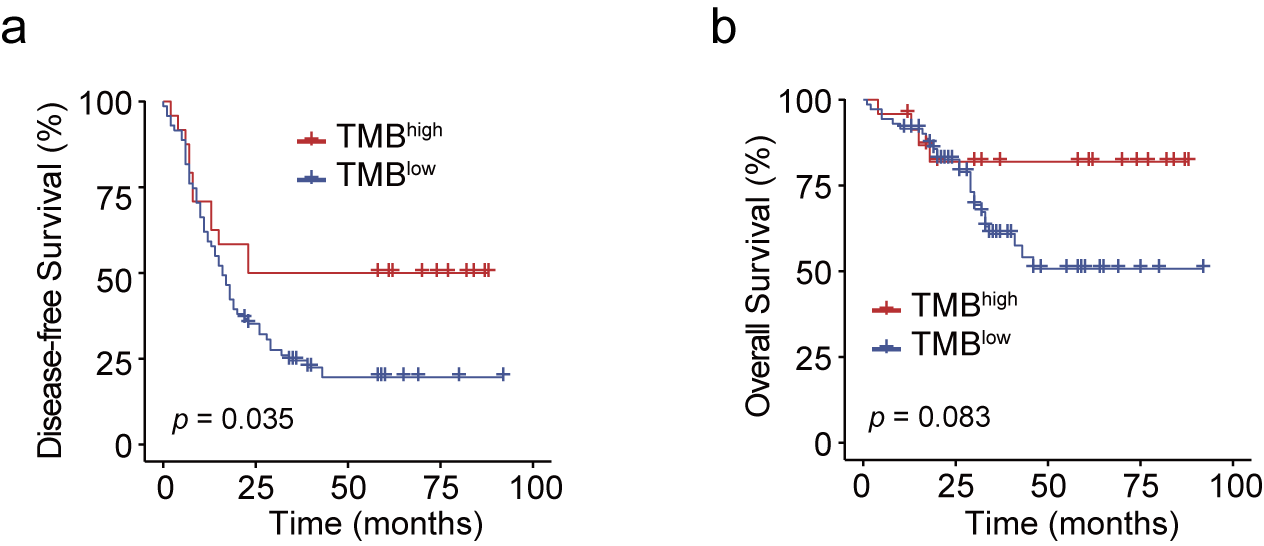

Supplement: Supplementary file 3 — Supplementary file3 (TIF 2040 KB) [file 43657_2024_158_MOESM3_ESM.tif]

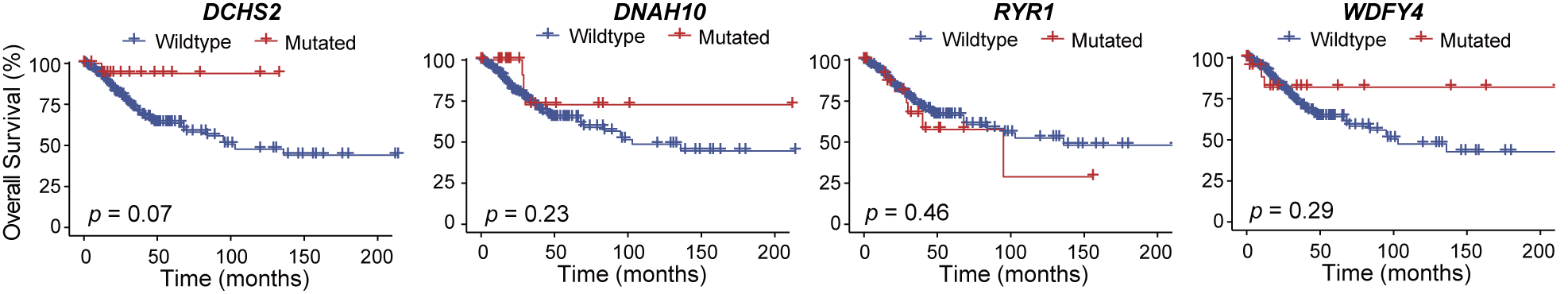

Supplement: Supplementary file 4 — Supplementary file4 (TIF 2155 KB) [file 43657_2024_158_MOESM4_ESM.tif]

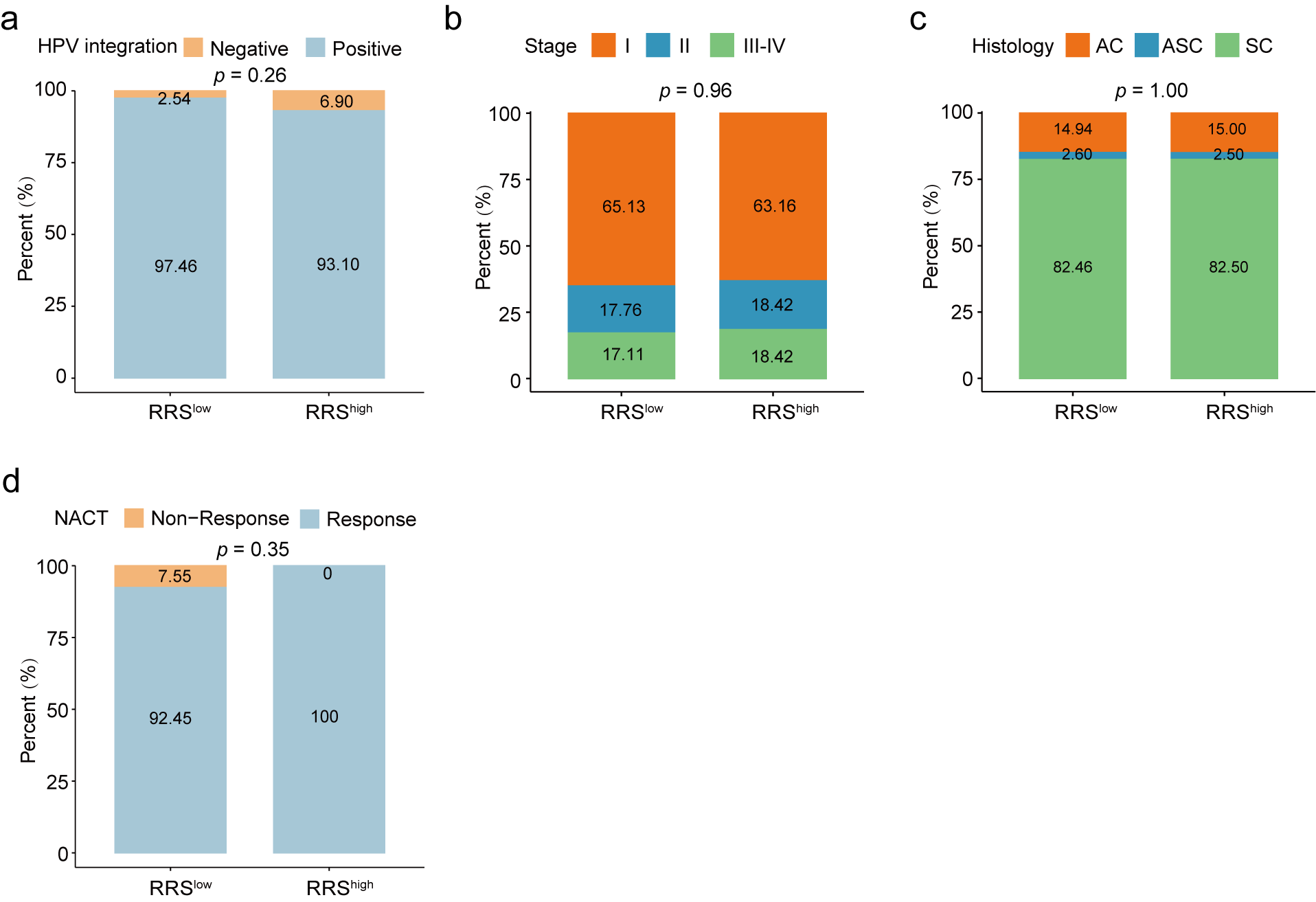

Supplement: Supplementary file 5 — Supplementary file5 (TIF 7395 KB) [file 43657_2024_158_MOESM5_ESM.tif]

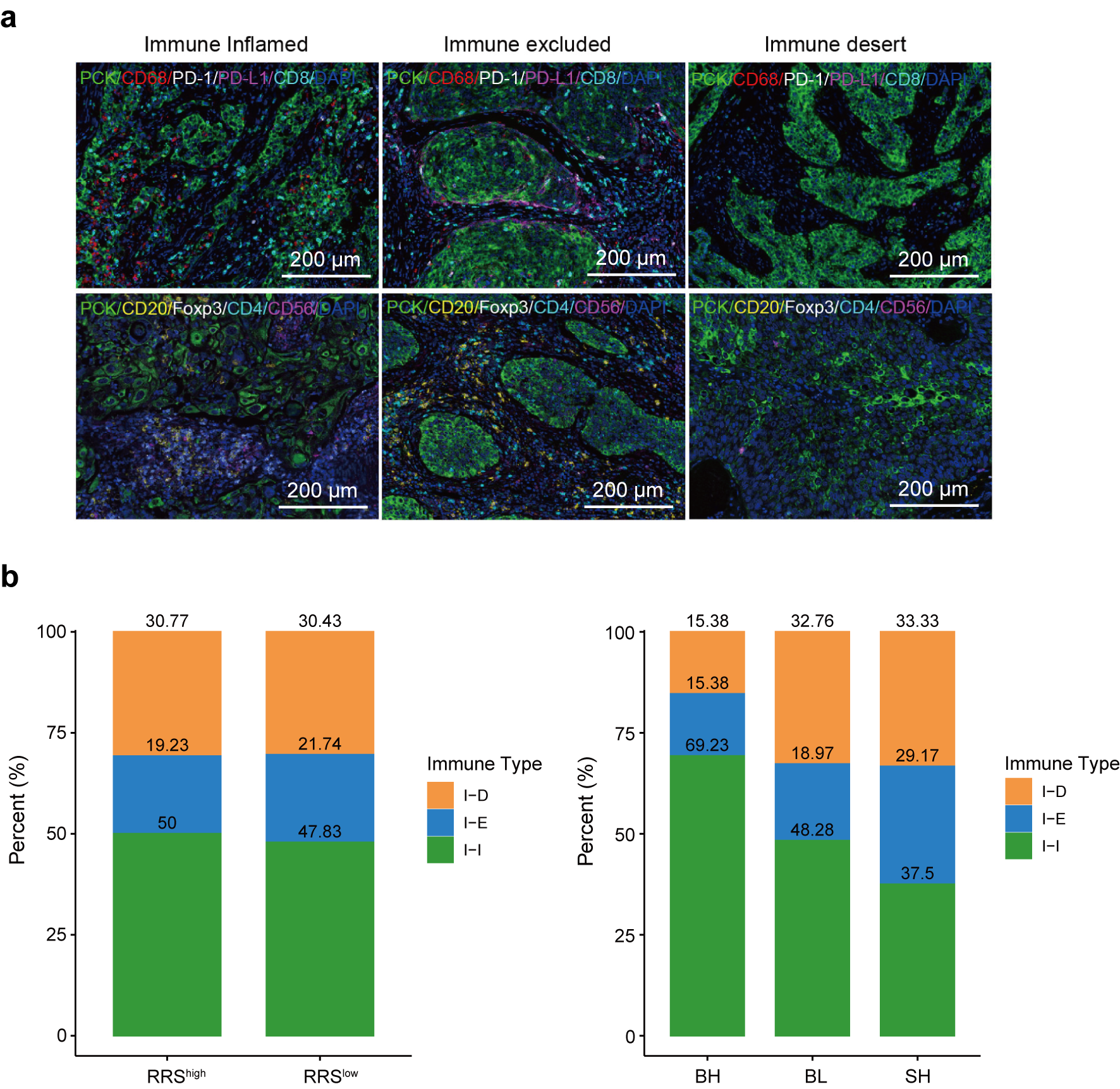

Supplement: Supplementary file 6 — Supplementary file6 (TIF 8986 KB) [file 43657_2024_158_MOESM6_ESM.tif]

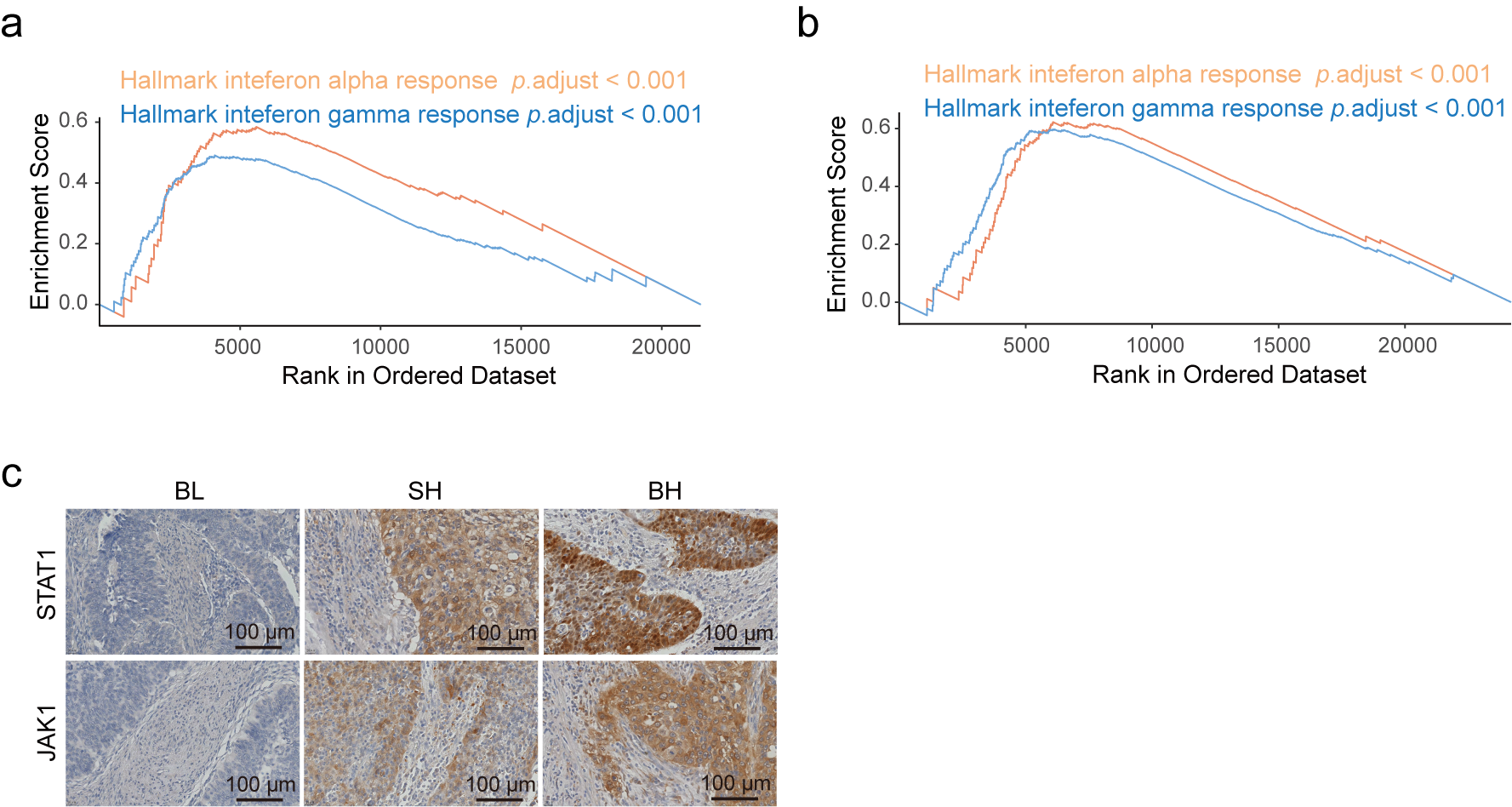

Supplement: Supplementary file 7 — Supplementary file7 (TIF 4959 KB) [file 43657_2024_158_MOESM7_ESM.tif]

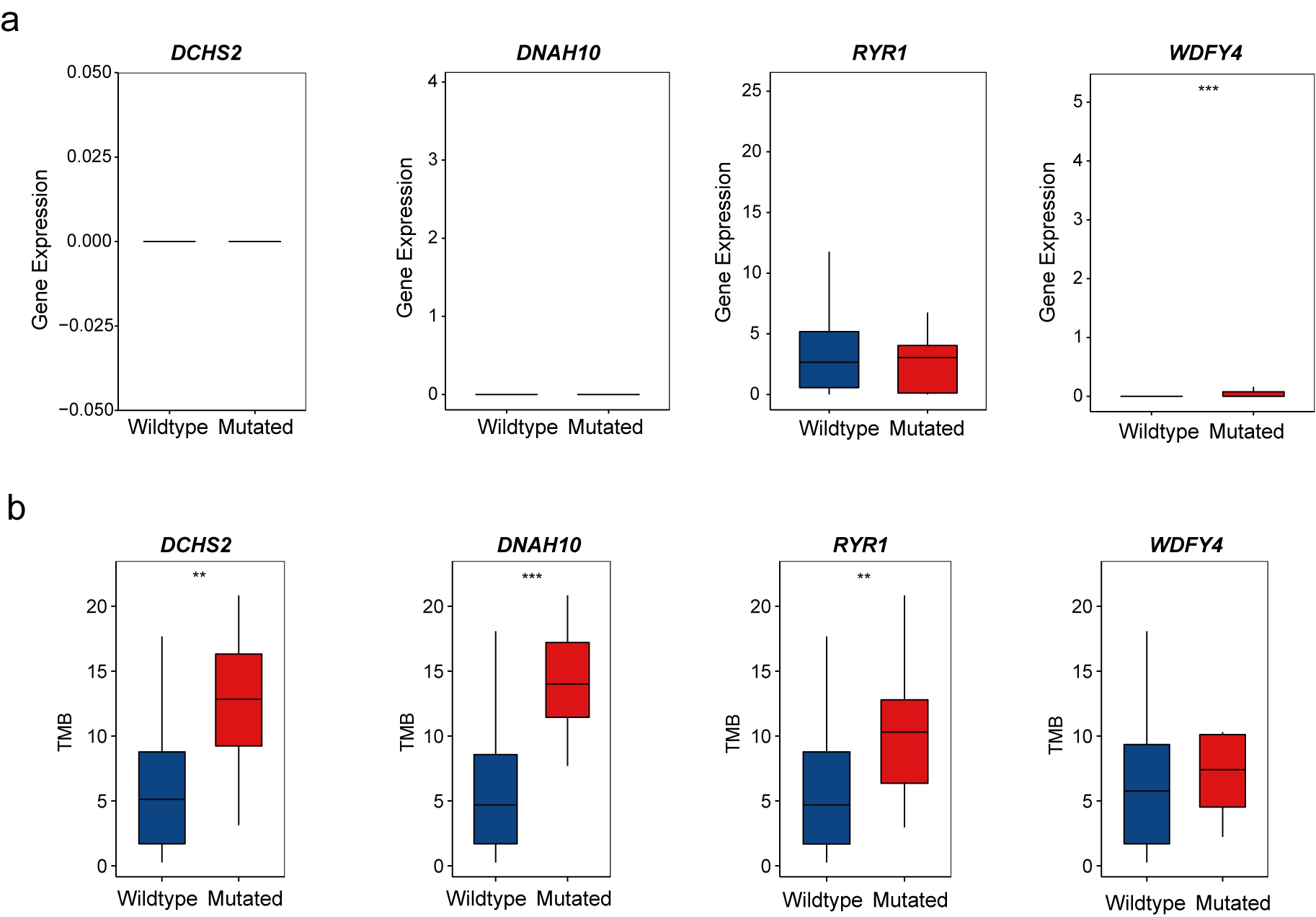

Supplement: Supplementary file 8 — Supplementary file8 (TIF 6829 KB) [file 43657_2024_158_MOESM8_ESM.tif]

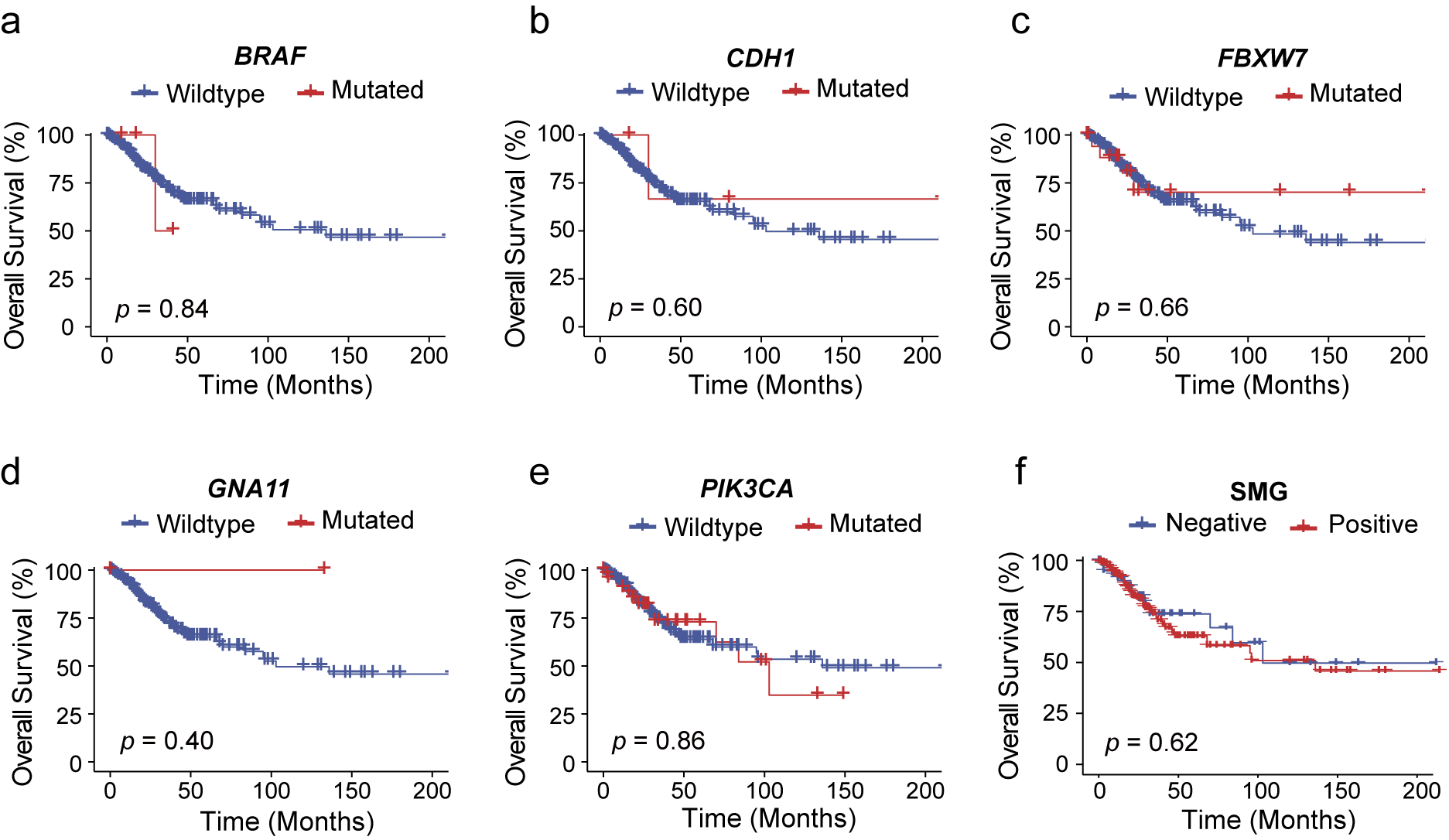

Supplement: Supplementary file 9 — Supplementary file9 (TIF 5989 KB) [file 43657_2024_158_MOESM9_ESM.tif]
